# Phloem exudate metabolic content reflects the response to water‐deficit stress in pea plants (*Pisum sativum* L.)

**DOI:** 10.1111/tpj.15240

**Published:** 2021-05-06

**Authors:** Sara Blicharz, Gerrit T.S. Beemster, Laura Ragni, Nuria De Diego, Lukas Spíchal, Alba E. Hernándiz, Łukasz Marczak, Marcin Olszak, Dawid Perlikowski, Arkadiusz Kosmala, Robert Malinowski

**Affiliations:** ^1^ Integrative Plant Biology Team Institute of Plant Genetics Polish Academy of Sciences ul. Strzeszyńska 34 Poznań 60‐479 Poland; ^2^ Laboratory for Integrated Molecular Plant Physiology Research (IMPRES) Department of Biology University of Antwerp Groenenborgerlaan 171 Antwerpen 2020 Belgium; ^3^ ZMBP‐Center for Plant Molecular Biology University of Tübingen Tübingen Germany; ^4^ Department of Chemical Biology and Genetics Centre of the Region Haná for Biotechnological and Agricultural Research Faculty of Science Palacký University Olomouc Czech Republic; ^5^ Institute of Bioorganic Chemistry Polish Academy of Sciences Noskowskiego 12/14 Poznan 61‐704 Poland; ^6^ Department of Plant Biochemistry Institute of Biochemistry and Biophysics Polish Academy of Sciences ul. Pawińskiego 5a Warsaw 02‐106 Poland; ^7^ Plant Physiology Team Institute of Plant Genetics Polish Academy of Sciences ul. Strzeszyńska 34 Poznań 60‐479 Poland

**Keywords:** abiotic stress, developmental plasticity, drought, phloem, oleic acid, *Pisum sativum*

## Abstract

Drought stress impacts the quality and yield of *Pisum sativum*. Here, we show how short periods of limited water availability during the vegetative stage of pea alters phloem sap content and how these changes are connected to strategies used by plants to cope with water deficit. We have investigated the metabolic content of phloem sap exudates and explored how this reflects *P. sativum* physiological and developmental responses to drought. Our data show that drought is accompanied by phloem‐mediated redirection of the components that are necessary for cellular respiration and the proper maintenance of carbon/nitrogen balance during stress. The metabolic content of phloem sap reveals a shift from anabolic to catabolic processes as well as the developmental plasticity of *P. sativum* plants subjected to drought. Our study underlines the importance of phloem‐mediated transport for plant adaptation to unfavourable environmental conditions. We also show that phloem exudate analysis can be used as a useful proxy to study stress responses in plants. We propose that the decrease in oleic acid content within phloem sap could be considered as a potential marker of early signalling events mediating drought response.

## Significance Statement

We describe the changes in phloem metabolic content of pea (*Pisum sativum*) plants subjected to drought and show how the observed patterns reflect physiological and developmental responses. Phloem exudate analysis itself gives us a clear and informative picture of the stress response strategy centred on above‐ground growth reduction and the reuse and redistribution of carbon and nitrogen. This provides evidence for the importance of phloem‐mediated long‐distance coordination in plant responses to drought. Fluctuations of particular metabolites in vascular sap can be used to monitor drought response in pea plants.

## INTRODUCTION

Water deficit has a large impact on the yield of *Pisum sativum* (pea) crops. Even mild and brief periods of drought stress affect important aspects, such as growth and internal trophic relations. As in other multicellular organisms, including humans, responses in particular organs have to be coordinated via the vascular system. The monitoring of vascular samples (blood) has long been established as an effective method to diagnose a wide range of conditions in human health (Coller, 2015). In plants this approach awaits further popularization as we lack effective tools and tests to determine plant physiological status based on phloem or xylem exudate content. The composition of phloem exudates is not constant and depends on the plant species, its development stage (Pate and Atkins, 1983) and the nutritional conditions (Tilsner et al., 2005). Phloem sap contains carbohydrates, sugar alcohols (polyols), amino acids, organic acids (Canarini et al., 2016), ions (Alfocea et al., 2000), phytohormones (Regnault et al., 2015), secondary metabolites associated with the stress response (Gowan et al., 1995), as well as macromolecules such as proteins (Malter and Wolf, 2011), RNA (Gamboa‐Tuz et al., 2018) and fatty acids (Barbaglia and Hoffmann‐Benning, 2016). The main form of sugars transported by phloem is sucrose (Lalonde et al., 2003), but other non‐reducing sugars are also present, such as raffinose, stachyose, verbascose, ajugose or sugar alcohols, such as mannitol and sorbitol (Canarini et al., 2016; Dinant et al., 2010). The possibility that phloem could be involved in the transport of reducing sugars had for a long time been discounted; however, in 2008 it was shown that glucose or fructose can be present in the phloem exudates of some plant species and constitute over 80% of the transported carbohydrates (van Bel and Hess, 2008). In addition to carbohydrates, the composition of phloem exudates also includes amino acids such as histidine, arginine, asparagine, glutamine, threonine, glutamic acid, proline, valine, methionine, isoleucine, leucine, phenylalanine or tryptophan (Canarini et al., 2016). Phloem exudates may also contain carboxylic acids that are Krebs cycle intermediates, that is, citric, isocitric, succinic, malic, fumaric and oxaloacetic acids (Canarini et al., 2016). Vascular exudate analyses have been applied successfully to study long‐distance coordination in plants at the RNA level (Kehr and Kragler, 2018) or to study movement of the RNA–protein complexes (Kehr and Kragler, 2018; Pahlow et al., 2018). The diversity of molecules representing core metabolic processes in phloem sap suggests that analysis of its composition may reveal a great deal of information concerning plant responses to stress. So far, the analysis of the compounds with ^13^C labelling in exudates has been useful in determining water‐use efficiency (WUE) and CO_2_ binding (Keitel et al., 2006). Moreover, it has been shown that leaf gas exchange measurements are very well correlated with carbon content in the phloem exudates (Merchant, 2012). If phloem sap composition reflects both the status of photosynthesis and cellular respiration it could be used to study stress adaptation mechanisms in plants. At present, however, there is a need to understand how changes in vascular sap reflect particular responses, therefore we have combined exudate metabolic studies with the monitoring of physiological and biometric parameters. Based on this approach, we have tried to elucidate how pea plants maintain their carbon and nitrogen balance during mild water deficit stress and to determine the contribution of vascular transport in the redistribution of these compounds. In addition, we have analysed phloem morphodynamics under these conditions; since drought triggers changes in the viscosity and water status of phloem sap, plants have to adopt certain strategies to avoid the scenario where stress completely compromises vascular transport (Sevanto, 2014). To understand how plants protect their conduits to maintain long‐distance coordination during abiotic stress, we have analysed phloem composition at the cellular level.

Our results show that plant responses to drought correlate with changes in phloem sap content, therefore we suggest that the exudate analysis method could be used as a diagnostic tool to search for drought‐tolerant genotypes.

## RESULTS

### Pea plants quickly adapt their growth and anatomy to water deficit conditions

**Figure 1:**
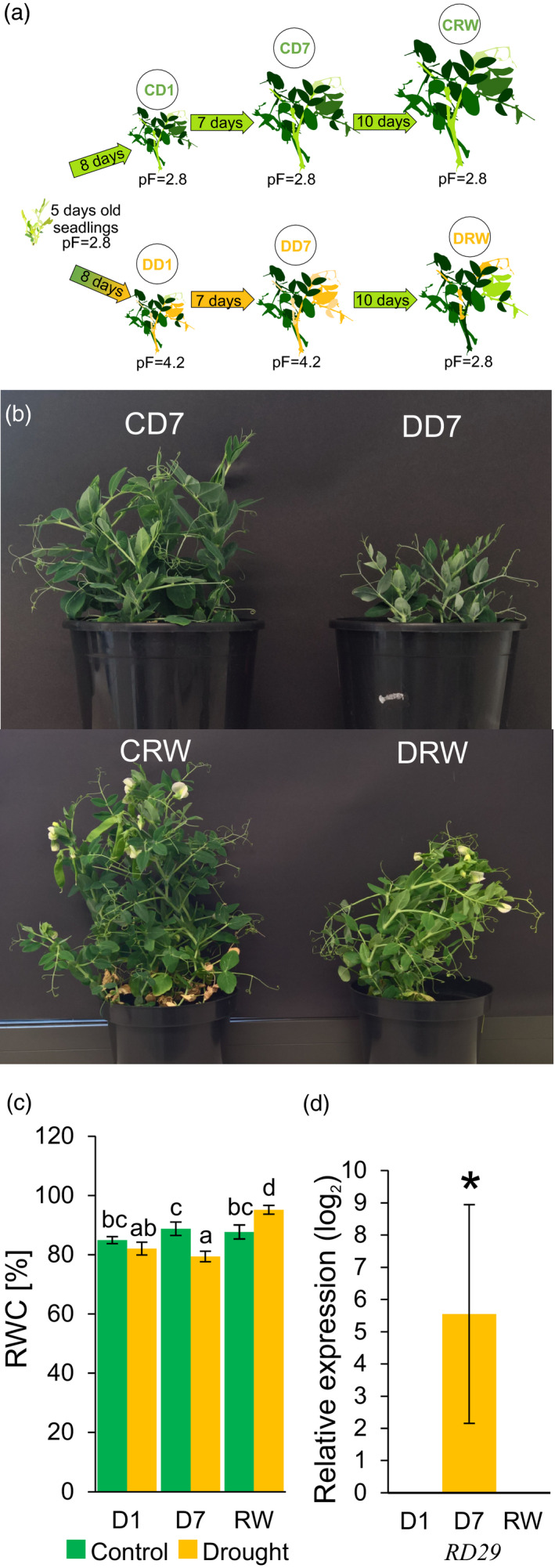
Experimental design and overall plant reaction to drought. Plants were subjected to stress by lowering the soil pF to 4.2 and appropriate samples were collected after 1 and 7 days of drought (DD1 and DD7) and 10 days after rewatering and adjustment of soil pF to 2.8 (DRW). Control samples were taken from plants cultivated under an optimal water regime of pF = 2.8 (CD1, CD7 and CRW). (a) Illustration of experimental design. (b) Photographs of representative plants showing the restriction of growth induced by drought. (c) RWC in fully expanded leaves; different letters indicate significant differences, as determined by analysis of variance (anova) with Fisher’s post‐hoc testing (*P* < 0.05). Error bars indicate the SEs (*n* = 7). (d) Expression of *PsRD29* in droughted leaves relative to controls. The asterisk indicates a significant difference (pairwise fixed reallocation randomization test, *P* < 0.001); error bars represent SEs (*n* = 3).

Drought has the most significant impact on the yield of pea when it occurs at the flowering stage (Andersen and Aremu, 1991). It has been found, however, that during the early vegetative stage drought can affect the morphology of the plant (in particular the position of the pod on a particular node), and therefore also the productivity of the plant (Klimek‐Kopyra et al., 2017). To gain more insight into the potential consequences of spring drought stress on peas, we have analysed changes in phloem sap content and evaluated their use as an indicator of physiological status. Five‐day‐old seedlings were subjected to a gradual decrease in water availability until the soil reached a pF status of 4.2, which was maintained for 7 days, followed by 10 days of a return to the optimal watering regime (Figure 1a). Morphological changes were monitored on the first and seventh day of pF 4.2 status (drought) and 10 days after rewatering (pF 2.8), and then compared with optimally watered controls (pF 2.8). We found that severe changes in the morphology of the above‐ground parts of plants can be observed already after 7 days of mild drought and plants could not recover their growth even 10 days after rewatering (Figure 1b). The relative water content (RWC) of fully expanded leaves decreased by 3.55% after 1 day and by 7.13% after 7 days of drought, compared with appropriate controls (Figure 1c). At 10 days after rewatering the RWC status increased to 7.44% compared with control plants of the same age (Figure 1c). The expression of a drought response marker gene *PsRD29* (Yamaguchi‐Shinozaki and Shinozaki, 1993) was highly upregulated (46.85‐fold increase, *P* < 0.001) at 7 days after drought stress (Figure 1d). Additional phenotypical characterization using spectral imaging with the same soil pF values, light intensity and temperature showed that shoot growth represented as the green area (Figure S1) and canopy perimeter (Figure S2) both decreased significantly. After rewatering, plants slightly increased their perimeter; however, they did not recover their green area size.

**Figure 2:**
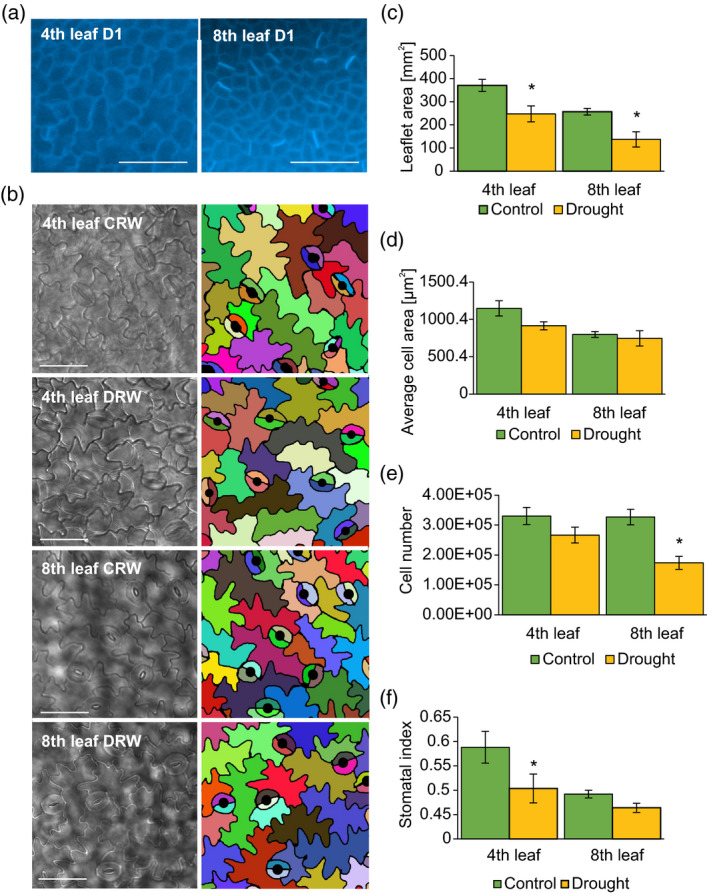
Anatomical changes in pea leaves depend on their meristematic status at the time of drought stress application. (a) Cell divisions in epidermal cells of leaf 4 and leaf 8 at day 1 (D1) of drought application, indicated by Aniline Blue staining marking callose deposition in newly created cell walls. (b) Abaxial epidermis of pea leaves at the indicated times. Pictures taken on cleared objects under Nomarski contrast. Scale bars for (a) and (b): 50 µm. Right panel represents the colour‐coded cell outlines used for further quantitative analysis: (c) leaflet area, (d) average cell area, (e) cell number, (f) stomatal index. Error bars represent SEs (*n* = 10). Statistically significant changes determined by anova and a post‐hoc Fisher’s test (average cell area and stomatal index) and Kruskal–Wallis test (cell number) are indicated with asterisks.

Organ growth reduction can be considered as an evolutionarily conserved mechanism used by plants to avoid unnecessary energy investment during adverse conditions. Modulation of leaf growth and leaf rolling mechanisms help to avoid water losses through evaporation (Avramova et al., 2015; Cal et al., 2019; Clauw et al., 2016). We found that even short periods of water deficit resulted in a decrease in leaf canopy. To develop a proper understanding of this response, we analysed the cellular composition of the abaxial epidermis in the second leaflet of leaves 4 and 8 following 1 and 7 days of drought. The choice of leaves was dictated by the lack (leaf 4) or presence (leaf 8) of meristematic activity within the epidermis at the time of drought application (Figure 2a). Samples for epidermal growth analysis were collected 10 days after rewatering when both leaves 4 and 8 have already completed their growth. After tissue clearing, we took pictures of the abaxial epidermis (Figure 2b) and analysed organ growth changes. We found that the leaflet area decreased upon drought in both leaves studied (Figure 2c). Cellular morphometry revealed that the observed changes had a different cellular basis. In leaf 4 a trend towards decreases in cell area and cell number, compared with controls, was observed but did not cross the threshold for significance (*P* = 0.06 and 0.07, respectively; Figure 2d,e). In contrast, in leaf 8 the cell number decreased significantly (Figure 2e), while the cell size was not affected (Figure 2d). On the other hand, the stomatal index decreased in leaf 4, whereas the observed decreases for leaf 8 were smaller and not statistically significant (Figure 2f). In conclusion, even mild and short drought stress induced leaf size reduction; however, particular changes in epidermal anatomy depend on the developmental context of cells at the time of stress application, with both cell division and expansion proving sensitive.

**Figure 3:**
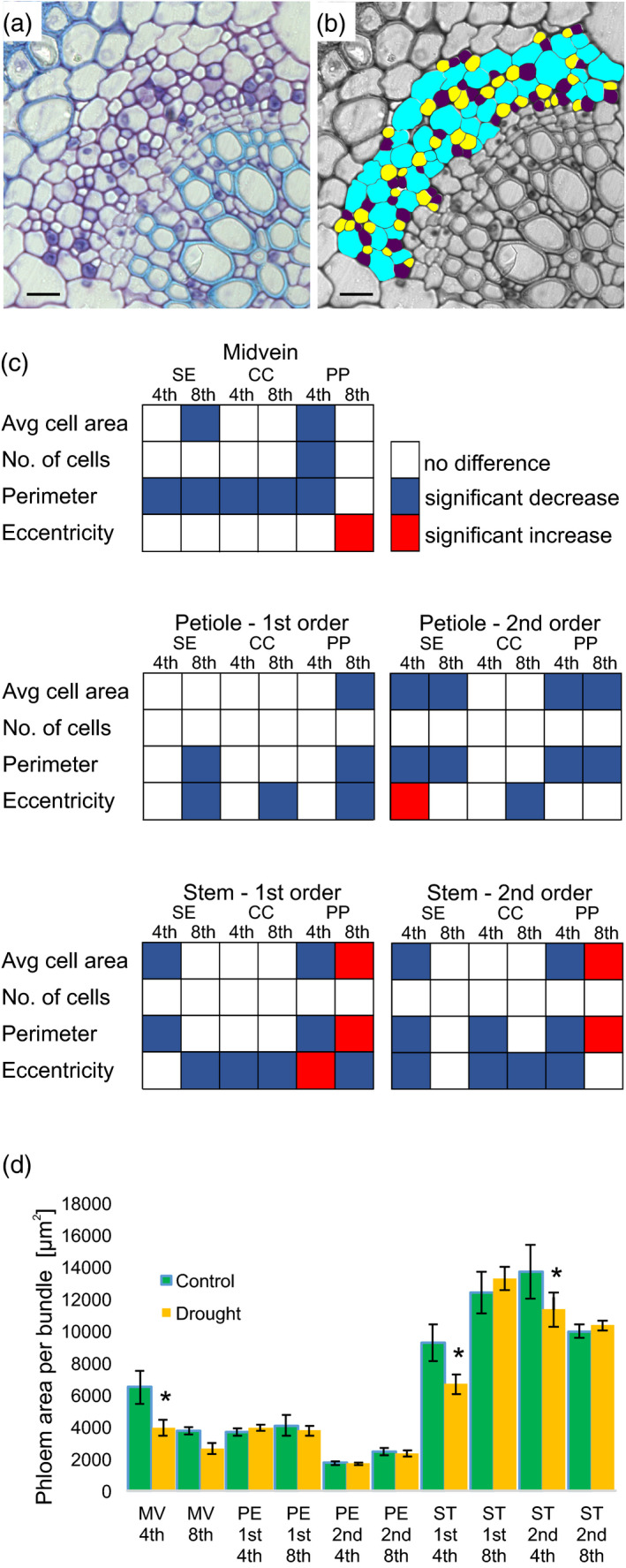
Drought affects the growth of phloem cells in *Pisum sativum* (pea). (a) Representative transverse section of pea vascular bundle stained with Toluidine Blue. (b) Grayscale picture with colour coding of particular phloem cell fractions used for scoring developmental plasticity within veins with lithographix: plum, companion cells (CC); yellow, sieve elements (SE); and cyan, phloem parenchyma (PP). Scale bars: 20 µm. (c) Summary of quantitative changes in phloem cell anatomy. White indicates no change, red indicates a significant increase compared with controls and blue indicates a significant decrease under drought (*P* < 0.05, determined by anova and a post‐hoc Fisher’s test). (d) Phloem area in conducting bundles in midvein (MV), petiole (PE) and stem (ST), calculated as the total area of all types of phloem cells. Error bars indicate SEs (*n* = 3, with five randomly chosen sections for each biological repeat).

Morphometric analysis revealed that the applied drought conditions did not affect phloem proliferation in the stems, petioles and midvein, with the exception of phloem parenchyma (PP), the number of which was reduced in the midvein of leaf 4 upon stress treatment. However, our analysis identified that drought triggers a whole spectrum of growth decreases in phloem cells (scored as the cell area, cell perimeter and cell eccentricity) (Figures 3, S3 and S4). These changes led to a decrease in the phloem area per bundle only in the midveins of leaf 4 and stem regions located below these organs (Figure 3d). PP are the most responsive cells in all studied organs, and cell area and cell perimeters are the cellular features that were most reduced by drought. It is interesting to note that PP area and perimeter increased in the first and second order of bundles of stems located below leaf 8, whereas the same parameter has decreased in bundles of stems located below leaf 4. Knowing that, at the time of drought stress application, leaf 8 is an actively dividing organ and a potential physiological sink, the observed changes may reflect an anatomical plasticity that facilitates nutrient redirection. It is also remarkable that phloem cell shape and growth, but not phloem cell proliferation, is affected by drought. Our biometric analysis has revealed another level of complexity that is related to vascular tissue differentiation. Namely, we observed a decreased number of bundles in petioles of leaf 8 subjected to drought, whereas in leaf 4 this phenomenon was not present. Moreover, the number of bundles in the stem section located below leaf 8 increased upon drought (Figure S5). We believe that this may be an important mechanism that helps to adjust the capacity of the vascular system to particular needs in times of energy restriction caused by drought.

### Pea plants modulate photosynthesis and respiration in response to mild drought

**Figure 4:**
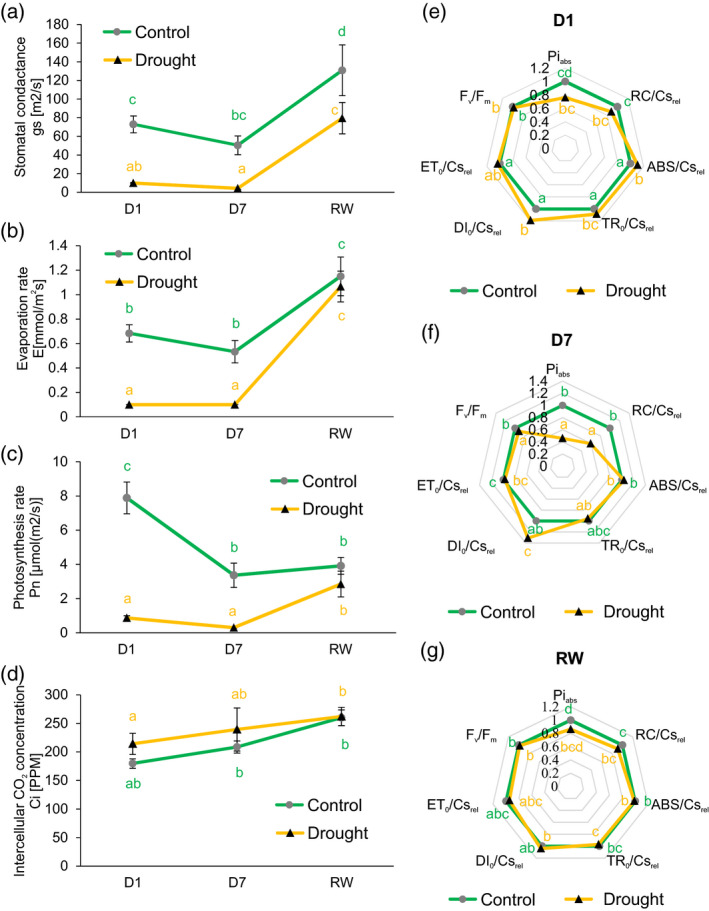
Changes in photosynthesis and gas exchange occurring in pea plants subjected to drought. Stomatal conductance (a), evaporation rate (b), photosynthesis rate (c) and intercellular CO_2_ concentration (d). Error bars indicate SEs (*n* = 6). Drought‐induced changes in electron transport were estimated by chlorophyll fluorescence measurements (*n* = 10; Pi_abs_, performance index for energy conservation from photons absorbed by PSII antenna in the reduction of quinone; RC/Cs_rel_, relative density of active reaction centres per leaf cross section; ABS/Cs_rel_, absorption flux per leaf cross section; TR_0_/Cs_rel_, relative trapped energy flux per leaf cross section; DI_0_/Cs_rel_, relative dissipated energy flux per leaf cross section; ET_0_/Cs_rel_, relative electron transport flux per leaf cross section; *F*_v_ */F*_m_, maximum quantum efficiency of PSII photochemistry) at day 1 (D1) (e), day 7 (D7) (f) and rewatering (RW) (g), presented as radar charts. The means and SEs of 10 replicates were calculated using anova and post‐hoc Fisher’s test. Different letters indicate a significant difference between means.

To understand how our experimental conditions affected the physiology of pea plants, we measured parameters relating to changes in solar energy conversion and subsequent carbon and nitrogen metabolism in plants. On the first day of drought plants already decreased their stomatal conductance (Figure 4a). This was further reflected by lower evaporation and photosynthesis rates (Figure 4b,c). Interestingly, these changes were not accompanied by a reduction of intercellular CO_2_ concentration (Figure 4d). Measurements of photosystem II (PSII) activity (Figure 4e,f) showed that its maximum photochemical efficacy (*F*_v_/*F*_m_) decreased after 7 days of drought. On the first day of stress PSII trapped more light energy relative trapped energy flux per leaf cross section (TR_0_/Cs_rel_); however, it did not influence the PSII electron transport chain relative electron transport flux per leaf cross section (ET_0_/Cs_rel_) and the collected energy was dissipated as heat relative dissipated energy flux per leaf cross section (DI_0_/Cs_rel_). The impact of drought on PSII was more pronounced on day 7 because a decrease in reaction centre density (RC/Cs_rel_) had occurred. Moreover, despite the fact that TR_0_/Cs_rel_ efficacy of light energy harvesting did not differ from the well‐watered control, the leaves were unable to sequester the energy productively and so it dissipated in the form of heat (DI_0_/Cs_rel_). After rewatering, all PSII parameters recovered (Figure 4g).

**Figure 5:**
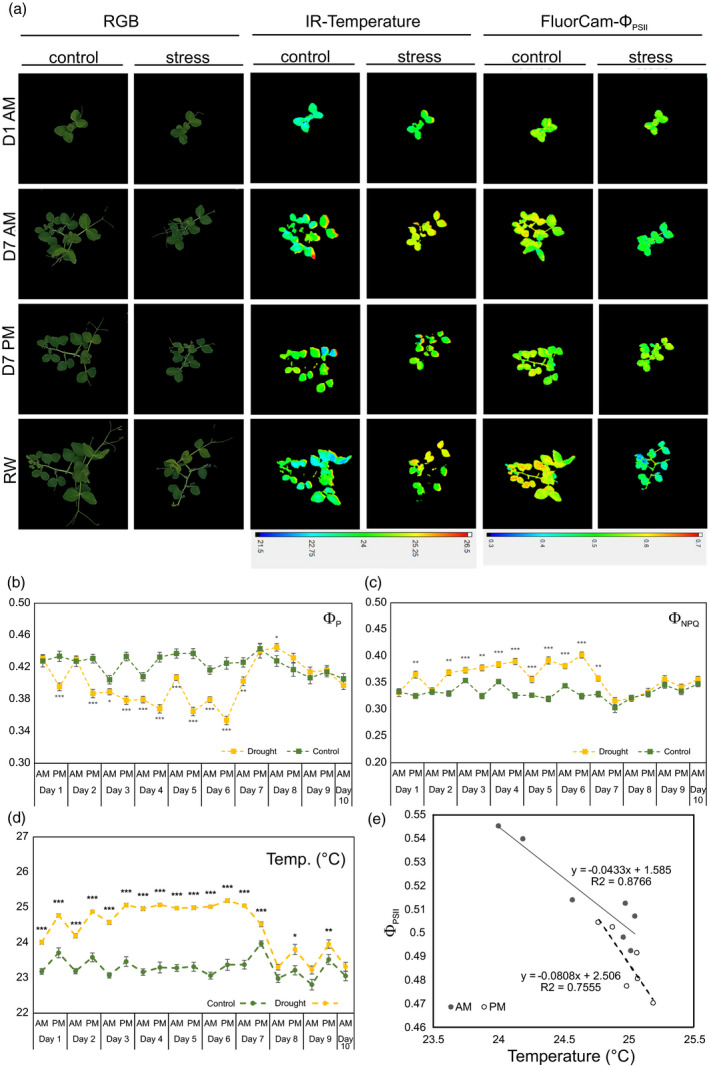
Drought‐induced changes in growth, heat dissipation and chlorophyll fluorescence in pea leaves monitored by RGB, IR and FluorCam imaging (a). Changes in the coefficient of photochemical capacity/quenching Φ_P_ (b), non‐photochemical quenching Φ_NPQ_ (c) and leaf temperature (d) upon drought stress. Values are means ± SEs (*n* = 18 for control and *n* = 30 for drought). Statistically significant changes determined by anova and a post‐hoc Fisher’s test are indicated with asterisks: *P ≤ 0.05; **P ≤ 0.01; ***P ≤ 0.001. Correlations of median parameters indicated light‐adapted maximum quantum yield of PSII (Φ_PSII_) and fluctuations in leaf temperature (e).

More detailed time‐course experiments carried out in the phenotyping system corroborated that the drought‐induced growth reduction was largely related to changes in chlorophyll fluorescence parameters. The light‐adapted maximum quantum yield of PSII (Φ_PSII_) and the coefficient of photochemical capacity/quenching (Φ_P_), describing the actual fraction of reactive centres being in the open state, were significantly reduced by drought stress (Figure 5a,b). At the same time, the Φ_NPQ_ parameter, describing non‐photochemical quenching that helps to dissipate heat, increased significantly (Figure 5c). The determination of leaf temperature by infrared (IR) imaging was even more sensitive, and significant differences were observed from the first point of the drought stress (Figure 5a,d). We also observed that changes in the median value of the fluorescence parameter Φ_PSII_ correlated with fluctuations in leaf temperature (Figure 5e). This was particularly apparent in the afternoon when the drought‐stressed plants were not able to cool down, thereby decreasing their fluorescence efficacy.

Together with biometric data these measurements were our reference for a greater understanding of changes in the metabolite content of phloem exudates.

### The content of pea phloem sap reveals drought‐induced responses and a tolerance that develops over the period of stress duration

**Figure 6:**
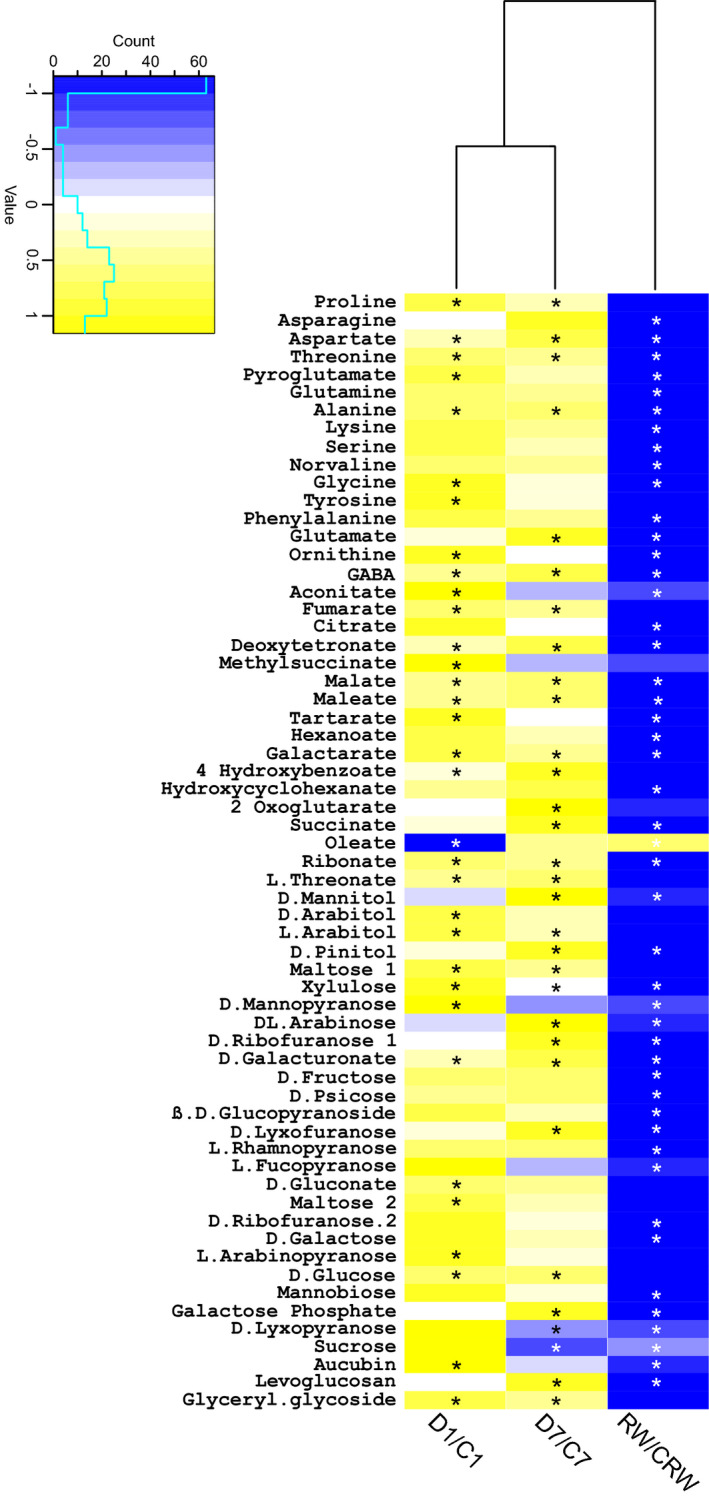
Differential accumulation of metabolites in phloem sap of pea plants upon drought and subsequent rewatering. The heat map presents the mean (log base 10) ratio between drought stress and controls, averaged over three biological replicates, with yellow representing increased abundance upon drought, blue representing decreased abundance upon drought and white representing no change. The stepped light‐blue line within the colour scale inset indicates the number of metabolites counted per level. Statistically significant changes marked with asterisks were calculated based on an analysis of variance (anova) with subsequent Fisher’s post‐hoc test at *P* < 0.05 (*n* = 3).

To identify metabolic changes underlying the reduction in plant growth observed upon water limitation, we have collected phloem sap exudates from excised leaves on the first and seventh day of drought and 10 days after rewatering, along with appropriate control combinations from plants growing under optimal water conditions. Samples were lyophilized and subjected to further GC/MS analysis and metabolite identification. To summarize the changes in phloem composition, we performed principal component (PC) analysis and projected the results onto a biplot representing the scores (variants as treatment and days) and loadings (metabolites). The first two PCs together captured 71.5% of the variance. PC1 (Dim1), which accounted for 55.2% of the total variation, separated the drought‐stressed plants from the controls and rewatered plants. Furthermore, PC2 (Dim2) separated the drought variants into two groups: (i) the plants on the first day of drought (DD1) were located with the metabolites (loadings) related to them; and (ii) the plants on the seventh day of drought (DD7) were located with their related metabolites (Figure S6). Upon drought and in response to rewatering (RW), 63 compounds exhibited differential accumulation in at least one time points (D1, D7 or RW) (Figure 6; Table S3).

**Figure 7:**
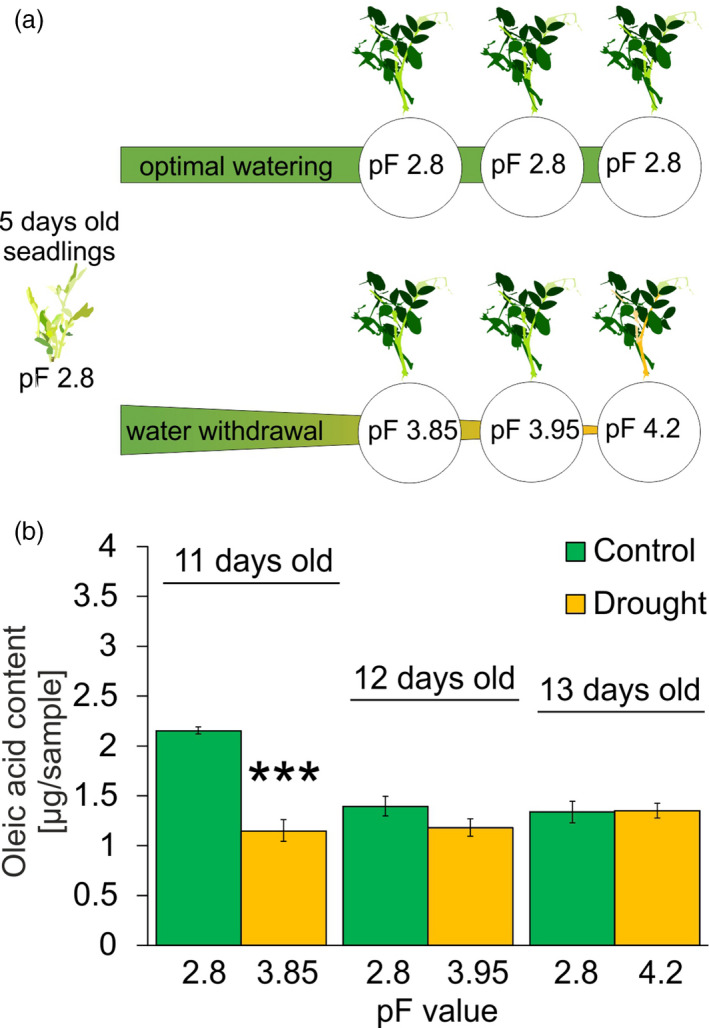
The decrease in the oleic acid content of phloem exudate occurs before soil reaches the wilting point. After water withdrawal phloem sap fractions were sampled when soil pF reached 3.85, 3.95 and 4.2 (critical wilting point). For each of these stages control exudates from optimally watered plants were also collected. (a) Illustration of the experimental design. The experiment was carried out in three independent biological repeats (*n* = 3); each consisted of exudates collected from four plants (with three randomly chosen fully developed leaves for each plant, which gives 12 leaves for each repeat). (b) Results of targeted analysis of oleic acid content. Statistical significance was determined by ANOVA, and post‐hoc Tukey’s honestly significant difference (HSD) test; asterisks represent statistically significant differences between optimal watering and water depletion combinations within the same time points (α = 0.05; *P ≤ 0.05; **P ≤ 0.01; ***P ≤ 0.001).

We observed a statistically higher accumulation of 32 metabolites at D1 and 29 metabolites at D7 after drought. The accumulation of only a few components decreased upon drought (at D1, oleic acid content decreased to non‐detectable levels, whereas at D7 the levels of lyxopyranose and sucrose decreased 3.12‐ and 6.61‐fold, respectively). The dynamics of oleate content change was further investigated in a separate experiment by targeted metabolomics in a time course spanning the period of water content decrease, leading up to the soil reaching the pF value of 4.2. Changes in this targeted experiment did not exactly match the picture obtained in non‐targeted experiments; however, again a very rapid decrease of oleate content in response to the reduction in water availability was observed (Figure 7).

After rewatering the majority of significantly differentially accumulating compounds decreased in the phloem sap of plants recovering from drought, compared with controls (46 compounds), whereas only one component increased (Figure 6; Table S3). Analyses of metabolite accumulation show that drought‐stressed time points are more alike than rewatered plants (Figure 6). The class of compounds predominantly accumulating in drought‐stressed plants were amino acids, followed by carbohydrates and organic acids. At D1 of drought, the accumulation of nine amino acids increased significantly: proline (30.53‐fold), ornithine (13.00‐fold), alanine (4.03‐fold), threonine (3.99‐fold), γ‐aminobutyrate (GABA; 3.31‐fold), tyrosine (3.09‐fold), aspartate (2.79‐fold), pyroglutamate (2.37‐fold) and glycine (2.21‐fold). At D7 of drought the phloem exudates had elevated accumulations of six amino acids: proline (12.18‐fold), glutamate (8.34‐fold), GABA (5.01‐fold), aspartate (4.46‐fold), alanine (3.33‐fold) and threonine (3.09‐fold). The carbohydrates and derivatives increasing in abundance at D1 of drought were: xylulose (19.77‐fold), l‐arabinopyranose (6.89‐fold), d‐arabitol (5.24‐fold), d‐glucose (5.23‐fold), maltose 1 (5.05‐fold), aucubin (4.88‐fold), gluconate (4.50‐fold), glyceryl glycoside (3.61‐fold), maltose 2 (3.52‐fold), l‐arabitol (3.23‐fold), galacturonate (2.88‐fold) and mannopyranose (2.21‐fold). The longer period of stress (D7) resulted in an increase of 13 components from this group, namely: galactose phosphate (24.22‐fold), d‐pinitol (7.31‐fold), xylulose (5.58‐fold), d‐galacturonate (4.43‐fold), d‐glucose (4.33‐fold), dl‐arabinose (3.95‐fold), maltose 1 (3.91‐fold), d‐mannitol (2.79‐fold), glyceryl glycoside (2.73 fold), l‐arabitol (2.49‐fold), d‐rybofuranose 1 (2.16‐fold) and levoglucosan (1.81‐fold). Conversely, the amount of d‐lyxopyranose and sucrose in phloem exudates decreased after D7 of drought, by 3.12‐ and 6.61‐fold, respectively. The 11 organic acids that accumulated in phloem exudates at significantly higher levels on D1 of drought, compared with controls, were: aconitate (8.17‐fold), deoxytetronate (7.37‐fold), hexanoate (5.97‐fold), tartarate (5.06‐fold), fumarate (4.67‐fold), threonate (4.59‐fold), ribonate (4.23‐fold), malate (2.38‐fold), methyl succinate (2.13‐fold), maleate (1.69‐fold) and 4‐hydroxybenzoate (1.29‐fold). Interestingly, at D1 of drought the content of oleate droped down to non‐detectable levels and was significantly lower than in control sap. After D7 of drought higher accumulations of 10 carboxylic acids were observed, namely deoxytetronate (8.95‐fold), 2‐oxoglutarate (6.81‐fold), threonate (4.45‐fold), hexanoate (3.80‐fold), fumarate (3.56‐fold), succinate (3.56‐fold), ribonate (3.10‐fold), malate (2.56‐fold), maleate (1.71‐fold) and 4‐hydroxybenzoate (1.31‐fold).

Different accumulation patterns were observed between D1 and D7, with some components increasing only at one time point, whereas others exhibited quantitative differences at both time points (Figure 6). For example, only at D1 was there a significant increase in glycine, ornithine, aconitate, d‐arabitol or aucubin, whereas at D7 higher amounts of the components pinitol, mannitol and glutamate are present in phloem sap. It was also interesting to see a higher accumulation of pyroglutamate at D1 versus increased glutamate at D7. Another interesting point is that in exudates collected at D7 after drought we can observe a significant decrease in sucrose levels, which is accompanied by increases in glucose and pinitol. These differences may in fact reflect a shift in the plant physiological status that occurs over time during the response to drought.

### Technical considerations related to metabolic studies of phloem sap

**Figure 8:**
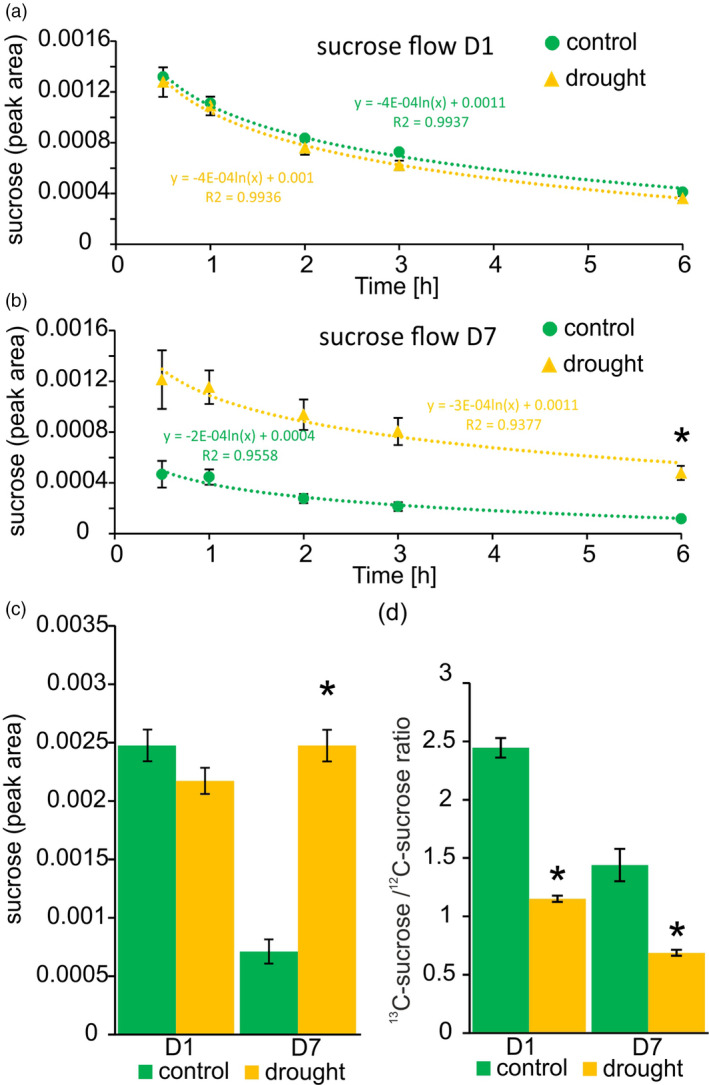
Detailed study of changes in sucrose content in the phloem sap of *Pisum sativum* (pea) plants subjected to drought. Velocity of sucrose exudation at day 1 (D1) (a) and day 7 (D7) (b) carried out for 0, 0.5, 1, 2, 3 and 6 h. Data are presented as the average values of normalized peak area reads characteristic for sucrose; error bars show SEs. The formula describing the exudation velocity is indicated by colour for each combination. (c) The sum of sucrose content in phloem sap collected as separate fractions in short intervals (0, 0.5, 1, 2, 3 and 6 h). (d) Proportion of labelled (^13^C) to non‐labelled (^12^C) sucrose based on peak area. Statistical differences were calculated by unpaired Student’s *t*‐test. Scale bars indicate SEs (*n* = 3; α = 0.05; *P ≤ 0.05). Each biological repeat consisted of exudates collected from four plants (with three randomly chosen fully developed leaves for each plant, which gives 12 leaves for each repeat).

Drought stress treatment leads to a decrease in water content, and therefore we can expect that some changes that we observed are the consequence of an increase in phloem sap viscosity. To some extent this phenomenon together with higher osmotic potential is an important mechanism that helps to retain water and to preserve phloem sap flow during times of water deficit (Sevanto, 2014). To determine how different viscosities affect the exudation velocity, we have performed a study in which samples were collected after 0, 0.5, 1, 2, 3 and 6 h of exudation and the sucrose content was quantified. We found that differences in exudation velocity between stressed and non‐stressed samples occurred within the first 3 h, but 6 h of exudation led to the collection of the majority of sucrose (Figure 8a,b). We can also conclude that the increased viscosity of phloem sap does not compromise sucrose exudation.

On the other hand, longer exudation times may also create a problem where some components degrade over time. Such a phenomenon was previously noticed in the case of sucrose stability (Tetyuk et al., 2013). Apparently, we also faced this scenario as higher levels of sucrose at 7 days after drought compared with representative non‐stressed controls were observed when exudation collection was performed over short periods (Figure 8c). We also re‐analysed glucose content in these the same exudates. We found that mean values for this component were higher in the phloem sap of drought‐treated plants; however, with the large differences between biological repeats, these changes were not statistically significant (Figure S7). These findings prompted us to perform an additional study that could at least partially address the origin of carbohydrates in the phloem sap of stressed plants. For this analysis, we used the ^13^CO_2_ isotope and traced sucrose with incorporated ^13^C. We found that in stressed plants a lower proportion of labelled to non‐labelled sucrose was present (Figure 8d). This is in agreement with the photosynthesis decreases observed in physiological measurements (Figure 4).

Another issue with the analysis of phloem metabolite content is the possibility of phloem sap contamination by xylem content. This is very difficult to estimate; however, it can be at least partially ruled out when exudates from the petioles are analysed. Based on our knowledge on the hydrodynamics of the vascular system, we can expect that when phloem exudates are collected from whole leaves, transpiration prevents leakage from the xylem, whereas exudates from petioles should also contain xylem components (Stroock et al., 2014). We have performed such a study and the metabolic petiole‐specific fingerprints did not correlate with the patterns observed in leaf phloem exudates. Figure S8 shows the distinct clustering, with dim1 accounting for 59.3% of the variance and dim2 accounting for 6.5% of the variance. We cannot rule out the possibility of contamination; however, this test does prove that the resulting metabolic fingerprints are based on changes in phloem sap.

Based on our velocity flow test, we can assume that longer exudation periods are more appropriate, especially when physiological stresses affecting phloem sap viscosity are the subject of study. The analyses presented, however, have shown that for some components (such as sucrose) additional inhibitors preventing degradation should be added. This is of course difficult when a non‐targeted analysis of the whole metabolome is performed, as numerous components are expected to be analysed. We found that sucrose degraded during exudation, and therefore we have to revise our previous observation and contend that the decrease in sucrose is an artefact related to the longer duration of sample collection.

## DISCUSSION

### Relationship between the drought‐triggered decrease in above‐ground growth and metabolic changes

Cell expansion in plants is driven by internal turgor pressure (Braidwood et al., 2014), therefore it is not surprising that limited water availability results in a decrease of growth. This developmental strategy, however, is not just a physical outcome of cellular water content but also a complex and finely adapted mechanism that enables plants to limit the loss of energy during difficult times. Cellular processes determining expansion (cell wall synthesis) or proliferation (cell cycle machinery) consume energy that will be scarce during times of stress. Confronted with drought stress, plants may deploy energy reserves to expand their root systems in search of new water resources or limit energy expenditure, and hence water use, to survive a temporary scarcity. Frequently, growth investments during a period of drought happen underground, whereas the upper part of the plant reduces expansion and its role is mainly to support the underground organs. Even a slight drop in the RWC of plants triggers long‐distance signal transduction (Kollist et al., 2019). In agreement with the central role of the phloem in the long‐distance coordination of numerous responses in plants (Takahashi and Shinozaki, 2019), our metabolomic studies showed that already after 1 day of drought the levels of aconitate, aucubin, ornithine, glycine, pyroglutamate, proline and GABA were increased. We speculate that this in turn triggers physiological responses aimed at conserving energy and the redistribution of carbon‐ and nitrogen‐containing components. We found that already by the first day of drought, stomatal conductance, the photosynthesis rate and the evaporation rate declined. This was not immediately reflected by a reduction in above‐ground growth, as the first significant decreases in plant canopy perimeter could only be seen after 5 days of drought (Figure S2). The observed physiological changes were the outcome of stomatal closure, which is a well‐known reaction to drought (Pirasteh‐Anosheh et al., 2016). It is intriguing that closing stomata did not lead to a decrease in intercellular CO_2_ concentrations. Partially this may be explained by the observed drop in the photosynthesis rate, which would diminish CO_2_ consumption; however, the higher production of metabolic CO_2_ during stress should also be considered. This suggests a scenario where plants decrease their water losses by limiting transpiration and try to cope with lower energy production (decrease in photosynthesis) through increased catabolism and carbon redistribution. This has been reflected by the higher accumulation of amino acids in phloem sap, perhaps being remobilized to physiological sinks like young leaves or roots from older leaves to compensate for the limitation in assimilate export. We have also found higher levels of glutamate, which plays a double role as a molecule that, after perception by glutamate receptor‐like ion channels, triggers the calcium ion signalling cascade, propagated over long distances through the phloem (Toyota et al., 2018), and (together with ornithine, proline and GABA) is a central component of nitrogen metabolism in plants (Majumdar et al., 2016). It is also worth mentioning that the so‐called ‘GABA shunt’ is a key pathway regulating carbon–nitrogen balance in plants (Michaeli and Fromm, 2015; Podlešáková et al., 2019). Furthermore, the increased accumulation of tricarboxylic acid cycle (TCA)/Krebs cycle components within the phloem of drought‐exposed plants suggests that pea plants facing water limitations may redirect metabolites that can be used to support primary metabolism in organs that are in need. In particular, we have found elevated levels of amino acids like proline (Pro), aspartate (Asp), threonine (Thr), alanine (Ala), glycine (Gly) and tyrosine (Tyr) that could be redirected towards actively dividing young organs during stress conditions to provide the components of the TCA cycle (Hildebrandt et al., 2015). We have also seen increases in other core components of the TCA pathway and interconnecting biochemical shunts allowing for alternative routes for the passage of carbon and nitrogen (Figure S8). These include *trans*‐aconitate, oxoglutarate, succinate, fumarate and malate (Rocha et al., 2010). Depending on the demand and oxygen availability, some of these components (e.g. oxoglutarate and succinate) can be incorporated into the main TCA cycle or, after conversion, used to provide ATP in the cells of actively growing organs. As droughted plants gradually decrease their growth, some changes observed in phloem sap at D7 after drought treatment may also arise from lower carbon consumption. Our biometric studies performed after rewatering showed that both the expansion and the proliferation of cells within leaves is affected by drought. During drought, numerous metabolites are redirected towards young and actively dividing leaves (Mundim and Pringle, 2018). This process however is not sufficient to support proliferation over longer periods of drought, therefore a decrease in cell number occurs. Such changes cannot be compensated for after rewatering as by that time meristematic activities cease. It has been shown that the decrease in the shoot/root ratio during abiotic stress is modulated over long distances by Pro synthesis or turnover (Sharma et al., 2011) and we found high levels of this amino acid in exudates from stressed plants. The higher levels of galactose phosphate detected in exudates after 7 days of drought also accords with the observed decrease in cell and organ size, as this component has been previously linked with the inhibition of auxin‐induced growth (Yamamoto et al., 1988). That change is also accompanied by a significant decrease in sucrose in phloem sap, which is known to regulate plant organ growth at multiple levels, including long‐distance coordination (Kircher and Schopfer, 2012). After verification, we found increased levels of sucrose in the exudates from stressed plants (Figure 8c). However, our further studies with ^13^CO_2_ supplementation have shown that a larger fraction of sucrose accumulated in stressed plants has no ^13^C incorporated (Figure 8d). That suggests the situation when carbohydrate reserves are remobilised in response to water deficit and decrease in leaf photosynthetic and gas exchange activity. In this case, even the higher levels of sucrose accumulating in phloem sap will not be directly translated into higher organ growth. A decrease in lamina size has obvious advantages because it reduces surface evaporation and helps to avoid light‐induced damage to photosystems. Frequently drought conditions coincide with light excess; however, in our experiments, conducted in controlled conditions, the light energy was the same for control and stressed treatments. Despite this fact, we could observe that drought stress itself induced non‐photochemical quenching to dissipate heat. Most probably this was not enough to fully protect the photosynthetic machinery and stressed plants were dissipating the energy by external heat emission. Larger leaf lamina and overall canopy size would additionally increase the impact of drought on the first phase of photosynthesis and make the maintenance and protection of photosystems more difficult. Our study shows that long‐distance coordination and phloem‐mediated change in the allocation or recycling of components that facilitate energy remobilization contribute to a drought‐avoidance strategy based on decreased above‐ground growth. The overview on changes in primary carbon and nitrogen metabolism observed in pea phloem exudates is presented in Figure S9.

### Phloem transport integrity during limited water availability

Vascular tissue in plants shows a wide range of developmental plasticity upon environmental stress (Figure 3). Changes in the vasculature affect the efficacy of phloem and xylem transport. As xylem plays a major role in water uptake and distribution, numerous studies describing the correlation between its particular anatomical features and parameters like hydraulic conductance and the likelihood of embolism formation have been conducted (Sack and Scoffoni, 2013). Phloem tissue is connected with xylem and therefore in response to changes in water potential triggered by drought, plants launch actions that aim to maintain the osmotic balance between these two vascular tissues (Sevanto, 2018). Plants intensify sugar loading to phloem that, along with the regulation of stomatal closure, is an important mechanism in regulating internal water relations. Sugar metabolism is very sensitive to stress conditions (Lemoine et al., 2013), and therefore it is not surprising that we have observed significant change in the abundance of particular carbohydrates within the phloem sap of pea plants subjected to drought (Figure 6; Table S3). Carbohydrates are tightly linked to basic cellular processes like photosynthesis and respiration, which are usually influenced by stress conditions (Figure S9). Initially we found that at D7 the sucrose content was decreased; however, this observation has been re‐evaluated after further targeted study and time‐course collection. We found that the amount of sucrose in the phloem increases, but that the long duration of phloem sap collection resulted in sucrose degradation (Figure 8b). This observation is in agreement with previous experiments performed by Tetyuk, Benning and Hoffmann‐Benning (2013), where similar effects of exudation time on sucrose stability have been observed. After the completion of all experiments, we can state that drought leads to the increased accumulation of sucrose, glucose and sugar alcohol (pinitol, arabitol and mannitol) in the phloem sap of pea plants. This elevates phloem sap viscosity and helps to maintain the high osmotic potential within the vasculature that prevents water loss and transport discontinuity during periods of low water availability. In addition, the content of Pro, which efficiently increases osmotic strength, was also elevated.

Plants have also adapted their phloem anatomy (Figure 3), possibly to avoid the scenario where prolonged drought increases phloem sap viscosity and disrupts the osmotic balance (Sevanto et al., 2014). To prevent decreases in the turgor pressure of phloem cells and potential blockages of assimilate transport, the sieve elements (SEs) and companion cells (CCs) are surrounded by parenchymatic cells that can stabilize and buffer turgor changes. Our detailed inspection of phloem anatomical changes triggered by drought has shown that, similar to tissues in other organs, cells in the vasculature grow less in response to water deficits. It is worth noting that the PP cells most strongly responded to changes in water availability. This is in agreement with their potential role in the optimization of water balance to protect vascular flow. Intriguingly, we have noticed that PP cells in stem regions located below the axis of leaf 4 and leaf 8 differentially responded to drought. The average PP cell area and perimeter increased in phloem bundles located in stems below the eighth leaf axis, whereas in PP cells of the same region below leaf 4 these same parameters were decreased. Aniline blue staining shows that, at the time of drought stress application, leaf 8 is an actively dividing organ. Cell proliferation requires a high‐energy investment that comes from major metabolic processes, including photosynthesis, mitochondrial respiration and protein or carbohydrate turnover (Siqueira et al., 2018). Our phloem sap analysis shows that there is an intensive redirection of metabolites related to the TCA cycle as well as carbohydrates, which can act as a source of energy. In plants, nutrients are delivered to rapidly growing organs from roots and older leaves (López‐Salmerón et al., 2019), therefore efficient carbohydrate transport via the phloem as well as the exchange between xylem and phloem of some amino acids (e.g. Arg, Asp or Glu), providing nitrogen to meristematically active tissues, must be secured. The observed developmental responses of PP cells to drought may be related to a particular physiological situation, where the plant needs to adapt phloem anatomy to facilitate lateral exchange between xylem and phloem and to maintain the flow of nutrients (especially nitrates) from the roots to rapidly growing organs (Aubry et al., 2019). Savage et al. (2013) have shown that newly developed phloem tissues mainly act to facilitate carbohydrate transport towards leaf primordia and young proliferating leaves requiring large energy investments, and moreover that the changes in carbon transport via phloem reflected the transition of true leaves from sink to source. These observations indicate that phloem‐driven carbohydrate transport is heterologous and depends on the developmental stage of the leaves. At present, we do not know the exact role of the observed differences in the drought‐induced developmental plasticity of PP cells in the stem regions underneath the proliferating leaf 8 and meristematically inactive leaf 4. At least partially it may arise from the different nutrient requirements of these organs. However, further understanding of the observed phenomenon needs more detailed experiments with labelled root‐derived nitrogen compounds or photosynthesis products of leaves.

### Vascular exudates as indicators of physiological responses in plants

The collection of molecules that move over long distances via vascular tissues provides a valuable insight into understanding how plants cope with adverse conditions. Phloem sap contains physiologically essential components like carbohydrates, amino acids, proteins or RNA molecules, and therefore changes in its composition may reflect particular plant responses. As mentioned by Dinant and Suárez‐López (2012), despite the potential usefulness of phloem sap collection methods they are artefact prone. The content of phloem exudates can be affected by phloem sap viscosity changes, degradation that occurs during exudation or possible contamination from xylem. On the other hand, phloem exudate collection approaches seem to be plausible for high‐throughput analysis of the long‐distance coordination of plant responses or for monitoring plant physiological status. In such studies, however, emerging patterns should be analysed rather than the change of a single component. In our work we used an ethylenediaminetetraacetic acid (EDTA)‐assisted method that helps to overcome the blocking of sieve tubes at the excision site by callose, thereby assuring efficient exudation (Tetyuk et al., 2013). We studied changes in the metabolic content of phloem sap, collected this way, that occur upon drought. Differential levels of some metabolites can be used in the future as potential indicators of the stress response. We have found that oleic acid content decreases in phloem rapidly upon water withdrawal. It has been shown that low levels of oleic acid promote nitric oxide (NO) synthesis and correlate with the upregulation of genes involved in NO‐mediated signalling (Mandal et al., 2012). We propose that the decrease in oleic acid within phloem sap could be considered as a potential marker of early signalling events that occur when pea plants are subjected to drought. Our study also shows that amino acid content as well as the accumulation of sugars and sugar alcohols in phloem sap reflects responses of pea plants to drought. The profiling of metabolic changes in phloem sap does not provide information establishing the origin and final destination of particular compounds. Such data can be obtained when isotope‐based approaches are applied (Gessler et al., 2004; Merchant, 2012). Our additional experiments where drought was applied in the presence of ^13^CO_2_ have shown that the proportion of labelled to non‐labelled sucrose was lower in stressed plants than in non‐stressed controls. This is in accord with observed growth reductions as well as physiological data showing that pea plants recalibrate various catabolic processes to cope with decreased photosynthesis and limited gas exchange. Our non‐targeted metabolomic studies provide an overall picture of the long‐distance mediation of drought responses in pea; this has proven to be very informative when combined with physiological measurements. Additional studies of the phloem content of other molecules like phytohormones, RNA or proteins may bring more detailed information regarding long‐distance signalling during stress response transduction (Buhtz et al., 2008; Giavalisco et al., 2006; Zhong et al., 1996). Based on our results, however, we conclude that changes in phloem sap amino acid metabolic profiles, as well as those of carbohydrates, can be used to evaluate the overall physiological reactions of plants to abiotic stress. We also propose that in the future this concept can be further explored for the development of diagnostic markers of stress responses in plants. Recent work presenting the use of a handheld near‐infrared (NIR) sensor for the determination of amino acid and lipid content included some aspects where oleic acid has been measured (Aykas et al., 2020). A properly developed sensor that allows for the rapid and non‐destructive estimation of oleic acid content in vasculature could be a valuable tool for breeders and commercial growers. This could also be an important component of future intelligent, water‐efficient, plant growth systems.

## EXPERIMENTAL PROCEDURES

### Biological material and growth conditions

Our experimental object was the green pea (*P. sativum* L.) cultivar ‘Walor’, developed for fresh consumption or processed canned pea production (PlantiCo Zielonki Sp. z o.o., https://plantico.pl). Five plants per pot were grown in pots filled with 0.5 kg of Klasmann No. 11 soil (pH 6.3) and were watered to achieve a soil moisture tension of pF 2.8, which has been proposed as an optimal water regime for pea (Kirkham, 2014). Plants were grown with 25°C days, 23°C nights in a 16‐h day/8‐h night cycle with 300 μmol m^−2^ sec^−1^ light intensity and a relative humidity of 40%. Optimal conditions were maintained for 5 days, then for the drought treatment watering was suspended until the soil moisture tension had reached pF 4.2, a critical wilting point for plants (Kirkham, 2014). The measurement of soil pF was performed with a ProCheck dielectric water potential sensor (MPS‐6; Decagon Devices, http://ictinternational.com). Drought conditions were maintained for 7 days with the soil moisture tension kept at the pF 4.2 value. After 7 days the droughted plants were returned to optimal conditions and supplied with water to maintain pF 2.8, this rewatering phase was carried out for 10 days. For control plants appropriate watering for pF 2.8 was maintained during the whole experiment. The time points selected for measurements and analyses carried out in this work were the first and seventh day of drought (D1 and D7, pF 4.2) and 10 days after rewatering (RW, pF 2.8). All appropriate control combinations were sampled at the D1, D7 and RW time points for plants grown under optimal water conditions throughout the whole experiment (Figure 1a).

For a deeper physiological characterization of pea responses under drought stress, plants were profiled with the OloPhen platform (http://www.plant‐phenotyping.org/db_infrastructure#/tool/57), equipped with integrated multiple sensors – top‐view red green blue (RGB) and thermal infrared (IR) camera and chlorophyll fluorescence imaging (FluorCam) – for non‐invasive analysis of plant physiological and morphological features. The experiment was performed following the same design as described above with minor modifications. For improved image analysis, one individual plant was grown per pot with at least 18 plants per treatment for a drought period of 7 days and a rewatering period of 3 days.

### Physiological measurements

Measurements of relative water content (RWC), gas exchange (GE) and various parameters of PSII activity were carried out on a fully expanded leaf (leaf 2) for the D1 and D7 time points. As a result of the fact that after rewatering leaf 2 was already beginning to senesce, the fully expanded leaf 4 was measured at this time point. For RWC, freshly cut leaves were weighed and subsequently incubated in water for 24 h in the dark, the weight of leaves at their maximal turgor was determined and the leaves were then desiccated for 48 h at 65°C to obtain dry weights. Then the RWC factor was calculated:

$$\text{RWC}\left(\%\right)=\frac{\text{FM}‐\text{DM}}{\text{TM}‐\text{DM}}*100\%,$$

where FM is the fresh mass of the leaf, DM is the dry mass of the leaf and TM is the turgid mass of the leaf.

Measurements were conducted on seven individual plants per treatment and sampling point. Gas exchange was measured at the D1, D7 and RW time points with the Ciras 2 portable photosynthesis system (PP Systems, https://ppsystems.com) for six control and six stressed plants growing in separate pots. Photosynthesis (Pn), evaporation (E), stomatal conductance (gs) and intercellular CO_2_ concentration (Ci) were determined. Measurements were performed under artificial light with an intensity of 150 µm of PAR m^−2^ sec^−1^, 25°C temperature, 380 ppm CO_2_ concentration, 100% humidity and a flow speed in the measuring chamber of 220 ml min^−1^. PSII photosystem activity was tested using a Pocket PEA device (Hansatech Instruments, http://www.hansatech‐instruments.com). For each combination and time point 10 measurements were performed. The parameters calculated are described in detail in Table S1.

The statistical significance of differences was determined by anova and post‐hoc Fisher’s test with statistica (Tibco, https://www.tibco.com). *P* < 0.05 was considered as a significant change. Image acquisition in the automatic phenotyping platform was carried out twice per day during the drought period and following rewatering using the RGB, IR and FluorCam sensors. The RGB image allowed the evaluation of shoot biomass growth as the change in number of green pixels (area). The kinetic changes in growth (canopy area and perimeter) were determined and the relative growth rate (RGR) related to area was calculated daily using the morning measurements:

$$\text{RGR}=\left[\text{ln}{\left(\text{green area}\right)}_{ti}‐\text{ln}{\left(\text{green area}\right)}_{ti‐1}\right]/ti‐\left(ti‐1\right),$$

where t*i* is time *i* (days).

The top‐view IR camera captures the heat signature of the plants, providing an indirect measure of stomatal conductance and transpiration (Sirault et al., 2009). Chlorophyll (Chl) fluorescence parameters were recorded using the top‐view FluorCam. A standard protocol was used for the measurement of Chl fluorescence quenching using the Chl fluorescence imaging (CFIM) protocols of the PlantScreen platform as described by Marchetti et al. (2019). Chl fluorescence parameters were calculated using fluorcam 7 (Photon Systems Instruments, https://psi.cz). The data obtained describe the quantum yield of PSII photochemistry in the light‐adapted state (Φ_P_), the quantum yield of regulatory light‐induced non‐photochemical quenching (Φ_NPQ_) and the maximal quantum yield of the PSII photochemistry for a light‐adapted state (Φ_PSII_ = (*F*_M_′ − *F*_0_′)/*F*_M_′).

### Biometric analyses

Images of cellular growth of the abaxial leaf epidermis were measured 10 days after rewatering on the second leaflet of leaf 4 and leaf 8. Cell images were acquired using a Zeiss Axio Scope A1 microscope (Zeiss, https://www.zeiss.com) under Nomarski’s differential interference contrast (DIC) contrast. Approximately 150–350 epidermal cells per leaf were outlined using a drawing tablet (Wacom, https://www.wacom.com) and imagej (Schneider et al., 2012). Individual cell areas were quantified with the use of the image analysis algorithm developed by Andriankaja et al. (2012). Average cell area was determined for each leaf analysed and the number of cells per leaf was estimated by dividing leaf blade area by average cell area. Stomatal index was calculated as a fraction of stomata in the total number of epidermal cells. For each experimental combination 10 leaves from randomly chosen plants were used. anova and the post‐hoc Fisher’s test were conducted to determine the statistical significance of changes in cell area and stomatal index, whereas cell number data were assessed with the Kruskal–Wallis test.

Cell divisions in the abaxial epidermis of leaf‐4 and leaf‐8 primordia at D1 of drought application were visualized with Aniline Blue staining performed as described by Kuwabara et al. (2011). Images were acquired under an M2 motorized Zeiss microscope equipped with a Colibri LED system, where fluorescence was excited with a 365‐nm LED and 4′,6‐diamidino‐2‐phenylindole (DAPI) Filter Set No. 49 was used to observe the Aniline Blue emission signals.

Phloem anatomy was determined for five randomly chosen sections from three biological repeats. Changes in midveins were scored for the second leaflets of leaves 4 and 8, the petioles and the stem regions located just below the axis of the analysed leaves from control and stressed plants 10 days after rewatering. Collected tissues were fixed in 4:1 98.8% EtOH/glacial acetic acid, embedded in Technovit 7100 (Kulzer, https://www.kulzer.com) and sectioned on a Leica RM2235 microtome (Leica Biosystems, https://www.leicabiosystems.com). For each area studied, three 10‐µm sections spaced 1 mm from each other were mounted on microscopy slides and stained with 0.05% Toluidine blue re‐suspended in a buffer containing 0.1 M Na_2_HPO_4_ and 0.05 M Na_3_C_6_H_5_O_7_. Images were acquired under an AXIO Image M2 microscope (Zeiss) equipped with a motorized stage and AxioCamICc5 camera. The morphodynamics of phloem were quantified using lithographix (de Reuille and Ragni, 2017; Sankar et al., 2014; Wunderling et al., 2016) and parameters such as number, area, perimeter and the eccentricity of cells were scored for the different phloem cell types (CC, PP and SE). The statistical significance of cell number change was evaluated with a Kruskal–Wallis test, whereas other parameters were assessed with anova followed by post‐hoc Fisher’s test. *P* < 0.05 was considered as significant.

### Quantitative gene expression

The *PsRD29* transcript sequence homologous to the Arabidopsis *RD29a* and *RD29b* genes (Yamaguchi‐Shinozaki and Shinozaki, 1993) was retrieved from the Pea RNA‐Seq gene atlas (Alves‐Carvalho et al., 2015), where it has been designated as *PsCam023410*. A 2‐µg portion of RNA isolated from leaf 2 at D1 and D7 and from leaf 4 at RW from stressed and control plants was used for the first‐strand cDNA synthesis performed with the Maxima First Strand cDNA Synthesis Kit (ThermoFisher Scientific, https://www.thermofisher.com). A 1‐µl volume of a fivefold dilution of this cDNA was used as a template for quantitative real‐time polymerase chain reaction (qRT‐PCR), carried out using the LightCycler 480 instrument (Roche, https://www.roche.com) and SensiMix SYBR No‐ROX Kit (Bioline, https://www.bioline.com). Primers were designed using quantprime (https://quantprime.mpimp‐golm.mpg.de/) (Table S2). Expression has been scored for three biological repeats and the levels obtained were normalized relative to the *PsUBC21* (*UBIQUITIN CONJUGATE 21*) gene (*PsCam043751*). Normalization was performed with rest‐384 2 (Pfaffl et al., 2002) and statistical significance was scored by the pairwise fixed reallocation randomization test.

### Phloem exudate isolation and metabolite analysis

Phloem sap was collected on the first and seventh day of drought and at 10 days after rewatering from whole leaves of stressed and control plants using the EDTA‐assisted method described by Tetyuk et al. (2013). Additionally, we performed control exudation from excised petioles on the first day. The experiment was carried out for three independent biological repeats and for each time point and treatment combination, exudates were collected from 12 randomly chosen fully developed leaves (three leaves from four plants for D1; four leaves from three plants for D7 and RW) that were placed in an Eppendorf tube containing 20 mm K_2_EDTA solution for 1 h in the darkness. After that solution was discarded and replaced with sterile water the exudate collection was carried out in the same conditions for 6 h, and samples were subsequently frozen in liquid nitrogen and stored at −80°C for further analysis.

Samples were lyophilized and subjected to derivatization with the *N*‐methyl‐*N*(trimethylsilyl) trifluoroacetamide (MSTFA). The GC/MS analysis was performed using an Agilent 7890A gas chromatograph (Agilent, https://www.agilent.com) connected to the Pegasus® 4D GCxGC‐TOFMS two‐dimensional gas chromatography time‐of‐flight mass spectrometer (Ouaked et al., 2003). A DB‐5 bonded‐phase fused‐silica capillary column (30 m in length, 0.25 mm inner diameter, 0.25 μm film thickness) (J&W Scientific Co., now Agilent) was used for separation. The GC oven temperature programme was as follows: 2 min at 70°C, raised by 10°C min^−1^ to 300°C and held for 10 min at 300°C. The total time of GC analysis was 36 min. Helium was used as the carrier gas at a flow rate of 1 ml min^−1^. One microliter of each sample was injected in splitless mode. The initial injector temperature was 40°C for 0.1 min and after that time raised by 600°C min^−1^ to 350°C. The septum purge flow rate was 3 ml min^−1^ and the purge was turned on after 60 s. The transfer line and ion source temperatures were set to 250°C. In‐source fragmentation was performed with 70 eV energy. Mass spectra were recorded in the mass range 35–850 *m*/*z*.

Data acquisition, automatic peak detection, mass spectrum deconvolution, retention index calculation and library search were performed using chromatof 4.51.6.0 (LECO, https://www.leco.com). To eliminate retention time (*R*_t_) shift and to determine the retention indexes (RIs) for each compound, the alkane series mixture (from C‐10 to C‐36) was injected into the GC/MS system. The metabolites were automatically identified by library search in Fiehn and National Institute of Standards and Technology (NIST) libraries (Kind et al., 2009; Yang et al., 2017). The analytes were considered to be identified when they passed a quality threshold: that is, similarity index (SI) above 700 and matching retention index ± 10. Artefacts (alkanes, column bleed, plasticizers, MSTFA and reagents) were identified analogously, and then excluded from further analyses. To obtain accurate peak areas for the deconvoluted components, unique quantification masses for each component were specified and the samples were reprocessed. All identified compounds were aligned using the statistical compare module of the chromatof package and then exported for further calculations. The profiles obtained were normalized against the sum of the chromatographic peak area using the total ion chromatogram (TIC) approach (Noonan et al., 2018).

The metabolomic data obtained were analysed using anova in statistica. For visualization, the data were also analysed using multivariate statistical analysis in r studio 1.1.463 (R Studio, https://www.rstudio.com) and for the heat map, the mean (log base 10) ratio between drought stress and controls was calculated.

To determine the potential involvement of oleic acid in early drought responses, phloem exudates were isolated from whole leaves of plants growing under control (pF 2.8) and progressive drought‐stress conditions (pF 3.85 and pF 3.95). Oleic acid content was estimated in a targeted analysis where chromatograms for selected ions of analysed compounds were plotted and their areas under the peaks were measured. These values were related to the area of the peaks plotted for the oleic acid standard (Supelco No. 75090) and analysed in known concentrations in their given range. This allowed for drawing calibration curves for these analytes and then absolute concentrations of compounds were calculated based on the least‐squares method.

### ^13^C labelling

To establish the origin of phloem‐located sucrose, ^13^CO_2_ feeding studies were performed in control and drought conditions. Microcentrifuge tubes containing 250 mg of NaH^13^CO_3_ (99 atom % ^13^C; Sigma‐Aldrich, https://www.sigmaaldrich.com) were attached to pots that were subsequently placed in plastic zip bags (with five plants in a single pot per bag) and additionally sealed with tape. Release of ^13^CO_2_ was triggered by the injection of 500 µl of saturated citric acid solution to the tubes. For each combination, samples were kept in this condition for 30 min followed by leaf excision and subsequent phloem sap collection across a time course of 0, 0.5, 1, 2, 3 and 6 h). Fractions of phloem sap were collected by moving detached leaves to fresh tubes containing water and immediately frozen in liquid nitrogen. After lyophilization and derivatization with MSTFA, samples were analysed by GC/MS as described above. The total level of sucrose was calculated by summing the intensities for all fractions, and the ratio of unlabelled to ^13^C‐labelled sucrose was determined (where sucrose molecules incorporating at least a single ^13^C were taken into account).

From this experiment we also calculated the sucrose velocity flow and presented it as the ratio of intensity observed in a particular measurement point to the time that passed from the start of the whole experiment.

## CONFLICT OF INTEREST

The authors declare that they have no conflicts of interest associated with this work.

## AUTHOR CONTRIBUTIONS

SB individually or in cooperation with other co‐authors performed all the experiments and she wrote the first draft of the manuscript. GB supervised and directed the work on leaf epidermis microscopy. LR supervised and directed the work on phloem anatomy. NDD, LS and AEH performed phenomics studies. ŁM performed the MS analysis of metabolites. MO assisted with some physiological measurements and helped to maintain plant growth. DP and NDD performed statistical data mining. AK helped to plan drought experiments and interpret the physiological data. RM designed and supervised the whole work, was involved in the data interpretation and wrote the final version of the article. The outcome of this work has been discussed by all the co‐authors and they all contributed in the preparation of the article.

## Supporting information

**Figure S1.** Above‐ground growth determined as change in green pixel area in RGB imaging. Error bars represent SE (*n* = 18 for control and *n* = 30 for drought). Statistically significant changes determined by ANOVA and a post‐hoc Fisher test are indicated with asterisks (*P ≤ 0.05; **P ≤ 0.01; ***P ≤ 0.001).

**Figure S2.** Above‐ground growth determined as green pixel‐based change in the perimeter. Error bars represent SE (*n* = 18 for control and *n* = 30 for drought). Statistically significant changes determined by ANOVA and a post‐hoc Fisher test are indicated with asterisks (*P ≤ 0.05; **P ≤ 0.01; ***P ≤ 0.001).

**Figure S3.** Phloem anatomy in plants subjected to stress and appropriate controls after rewatering.

**Figure S4.** Quantitative changes in phloem cell anatomy in response to drought. Error bars indicate SE (*n* = 3; 5 randomly chosen sections for each biological repeat). Statistically significant changes determined by ANOVA and a post‐hoc Fisher test (area, perimeter, eccentricity) and Kruskal‐Wallis test (cell No. / bundle) are indicated with asterisks.

**Figure S5.** Change in bundle number that occurs after rewatering in comparison with a representative control.

**Figure S6.** Principal component analysis showing a scatter plot for differentially accumulated metabolites (*n* = 3) at each time point on principal component 1 (Dim1) and principal component 2 (Dim2).

**Figure S7.** The sum of glucose content in phloem sap collected as separate fractions over short intervals (0, 0.5, 1, 2, 3 and 6 h).

**Figure S8.** Principal component analysis (*n* = 3) showing a scatter‐plot comparison of metabolite fingerprints obtained for phloem exudates from leaves (black) and from excised petioles (red).

**Figure S9.** Visualization of changes in selected metabolites of primary carbon and nitrogen metabolism in phloem exudates of drought‐treated pea plants at the represented times.

**Table S1.** Physiological parameters measured with Ciras 2.

**Table S2.** Sequences of nucleotide primers used for qRT‐PCR.

**Table S3.** Metabolite identification based on the Fiehn and NIST libraries (Kind et al., 2009; Yang et al., 2017).

## Data Availability

All relevant data can be found within the manuscript and its supporting materials.
