# Supplementary figures and images for: Phloem exudate metabolic content reflects the response to water‐deficit stress in pea plants (Pisum sativum L.)
Source: Plant J. 2021 May 6;106(5):1338–55. doi: 10.1111/tpj.15240 (PMC8360158; doi:10.1111/tpj.15240)

(a)

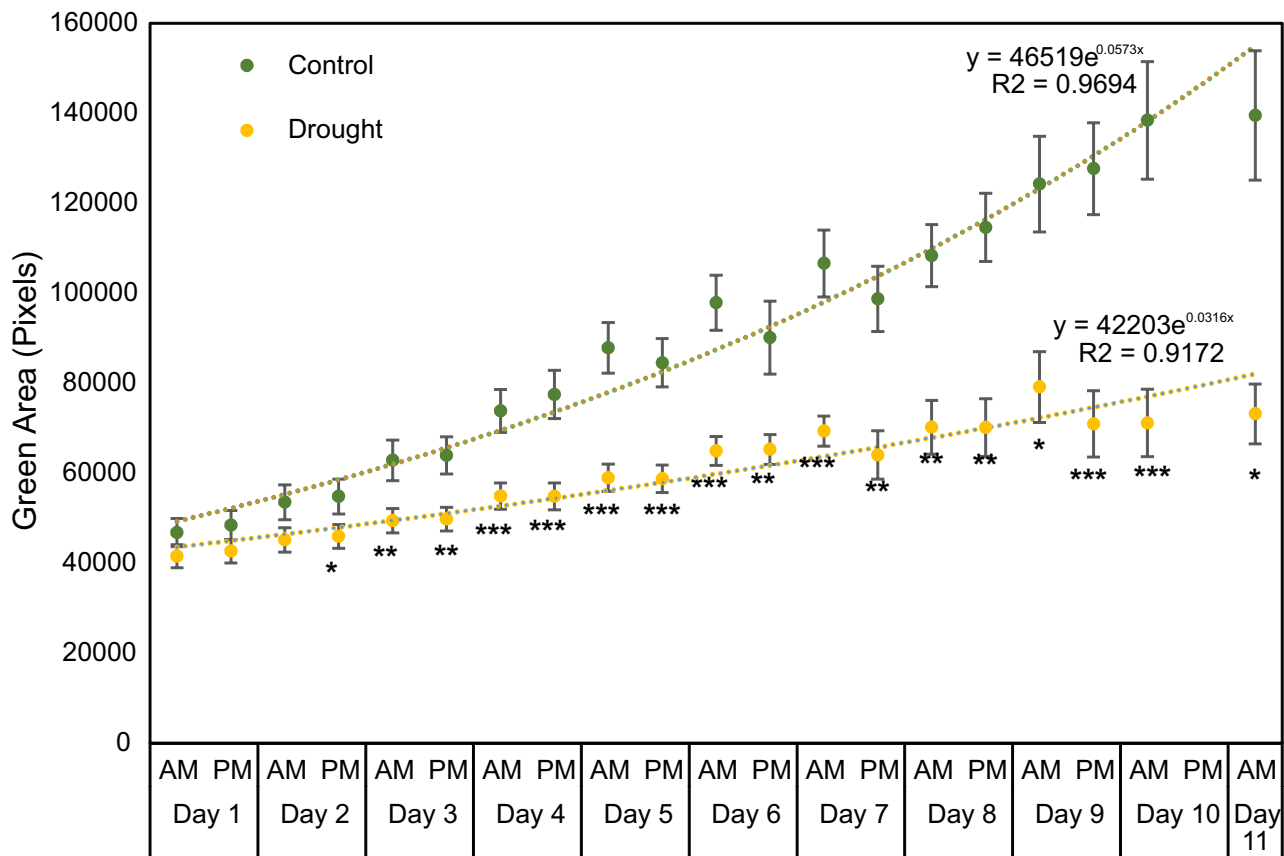

(b)

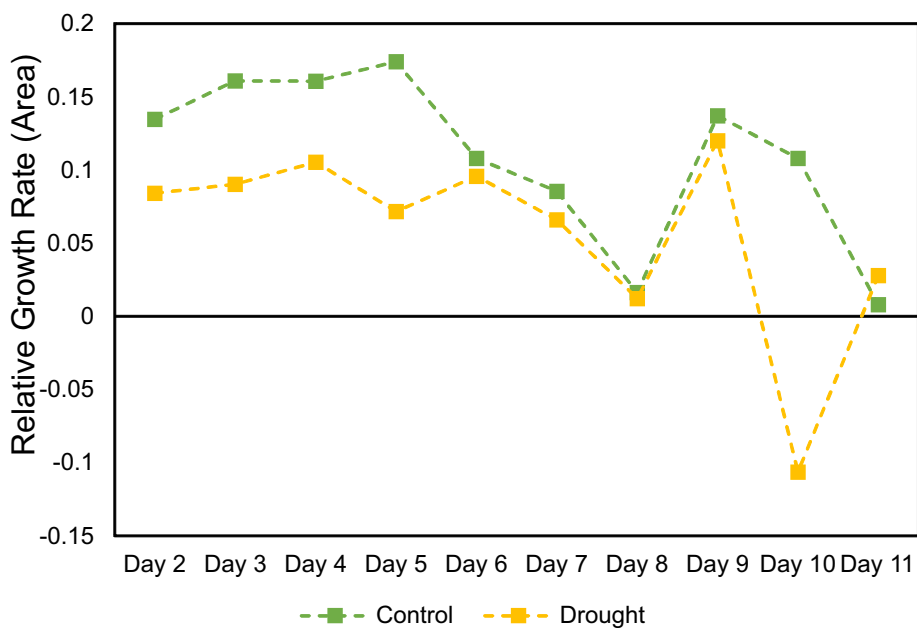

Supplement: Supplementary file 1 — Figure S1. Above‐ground growth determined as change in green pixel area in RGB imaging. Error bars represent SE (n = 18 for control and n = 30 for drought). Statistically significant changes determined by ANOVA and a post‐hoc Fisher test are indicated with asterisks (*P ≤ 0.05; **P ≤ 0.01; ***P ≤ 0.001). [file TPJ-106-1338-s008.pdf]

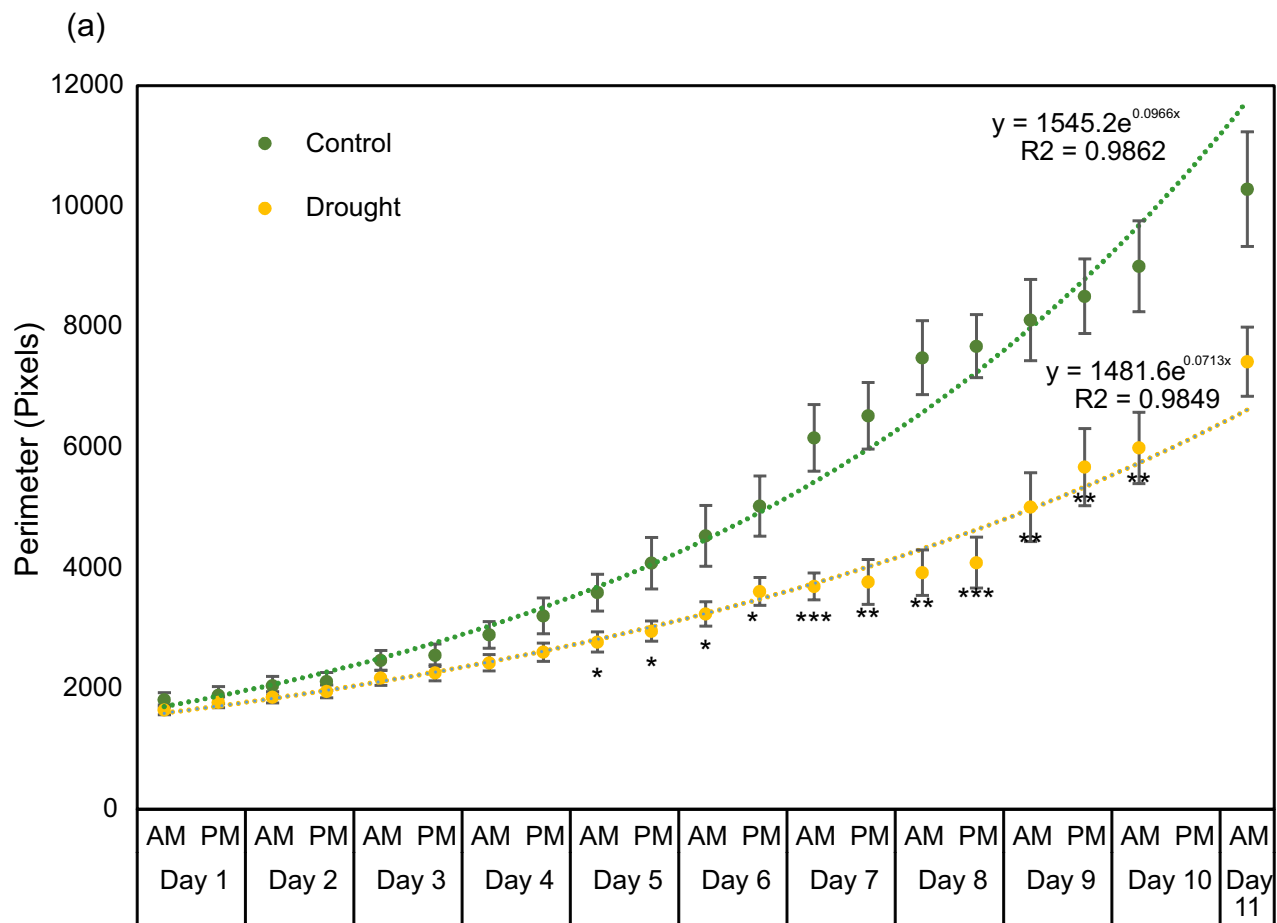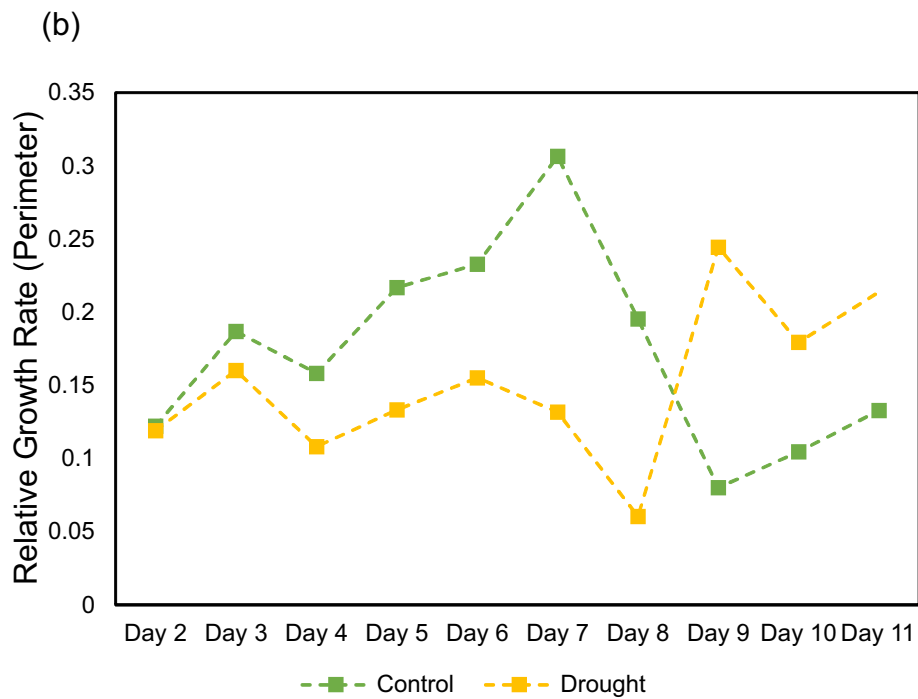

Supplement: Supplementary file 2 — Figure S2. Above‐ground growth determined as green pixel‐based change in the perimeter. Error bars represent SE (n = 18 for control and n = 30 for drought). Statistically significant changes determined by ANOVA and a post‐hoc Fisher test are indicated with asterisks (*P ≤ 0.05; **P ≤ 0.01; ***P ≤ 0.001). [file TPJ-106-1338-s006.pdf]

4th C

4th D

8th C

8th D

Midvein

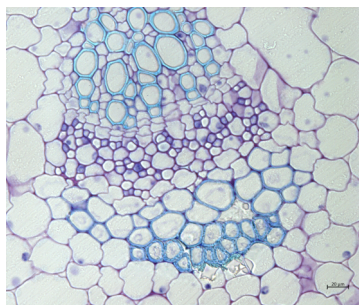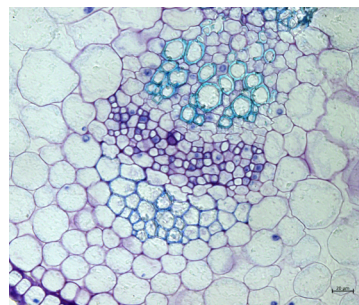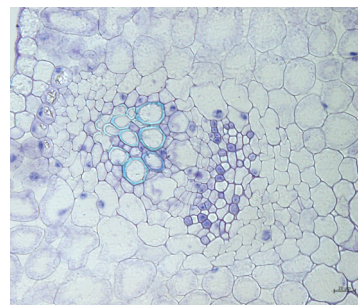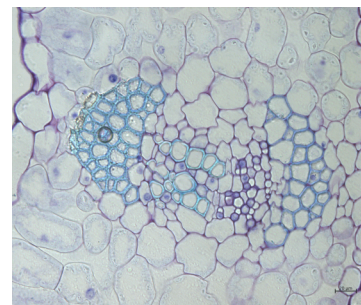Petiole  
1st order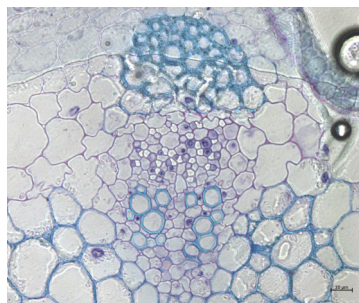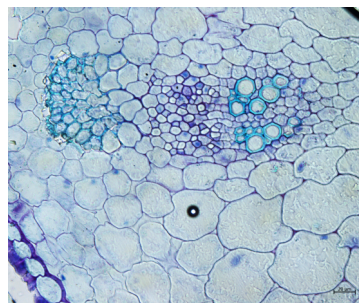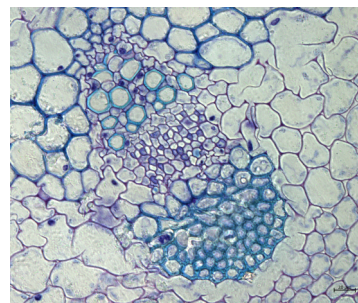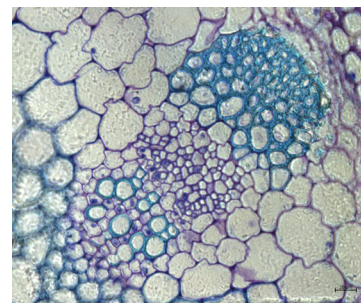Petiole  
2nd order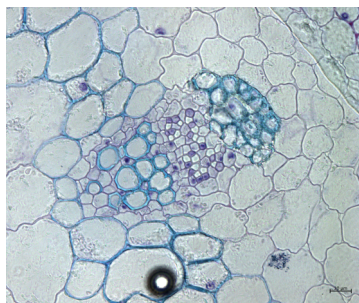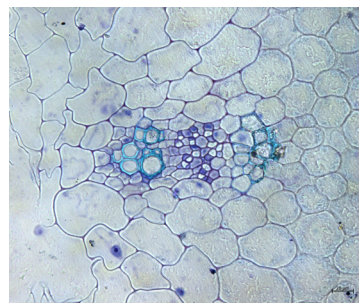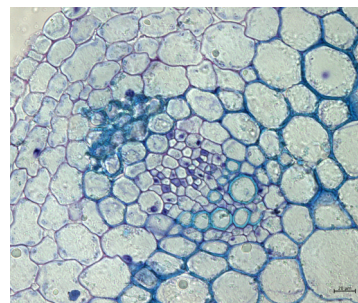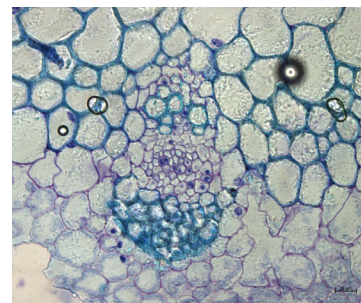Stem  
1st order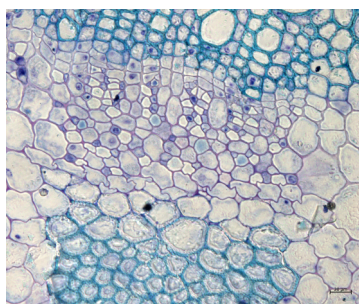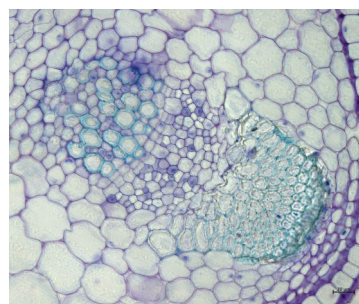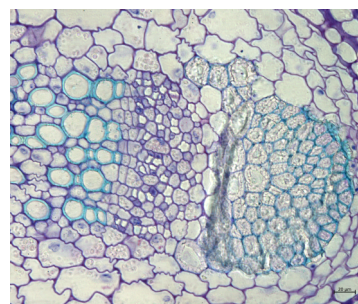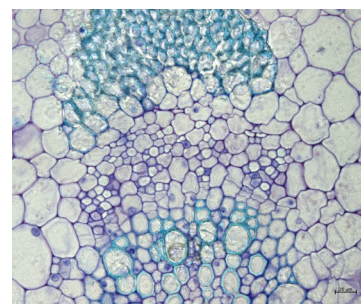Stem  
2nd order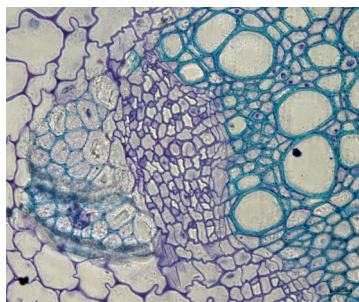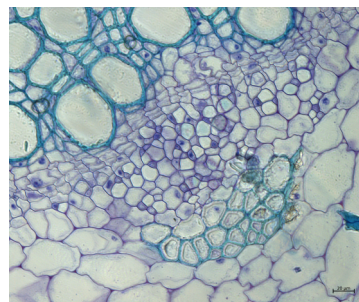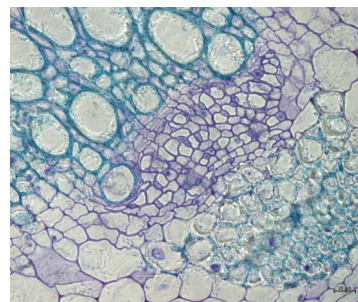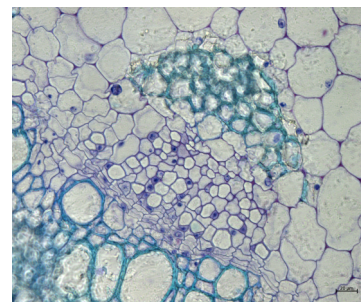

Supplement: Supplementary file 3 — Figure S3. Phloem anatomy in plants subjected to stress and appropriate controls after rewatering. [file TPJ-106-1338-s007.pdf]

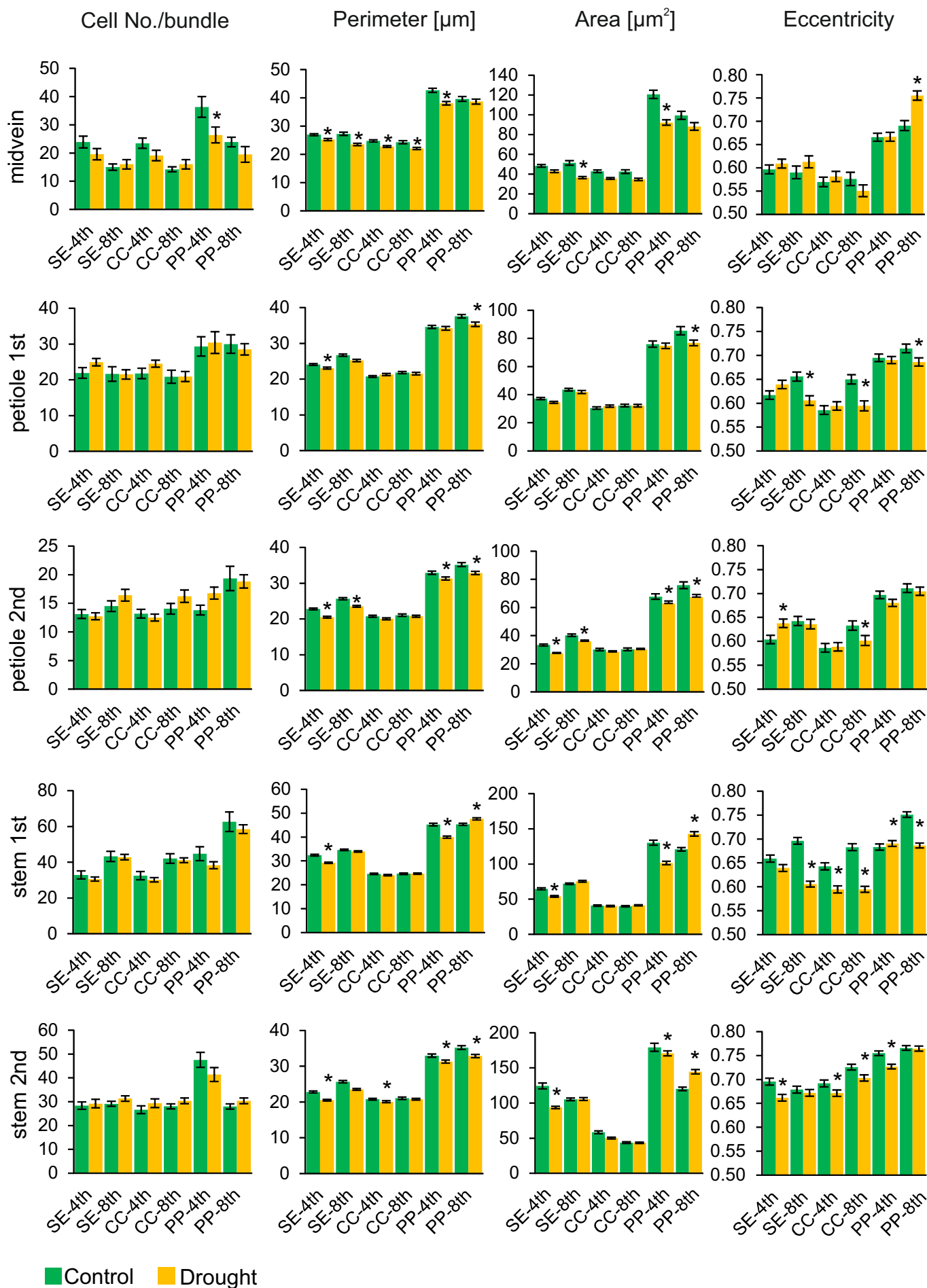

Supplement: Supplementary file 4 — Figure S4. Quantitative changes in phloem cell anatomy in response to drought. Error bars indicate SE (n = 3; 5 randomly chosen sections for each biological repeat). Statistically significant changes determined by ANOVA and a post‐hoc Fisher test (area, perimeter, eccentricity) and Kruskal‐Wallis test (cell No. / bundle) are indicated with asterisks. [file TPJ-106-1338-s003.pdf]

(a)

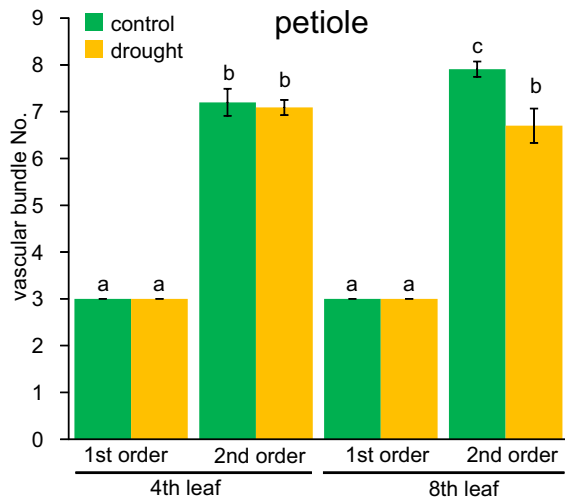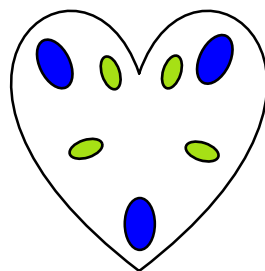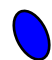

1st order bundle

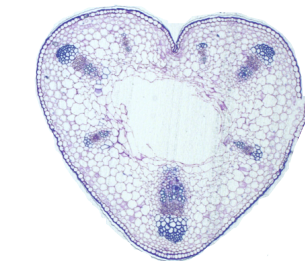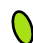

2nd order bundle

(b)

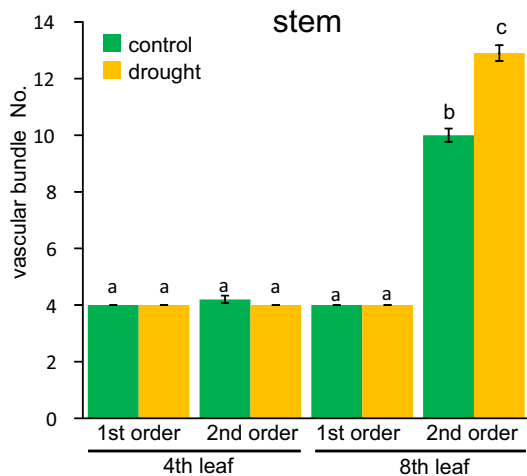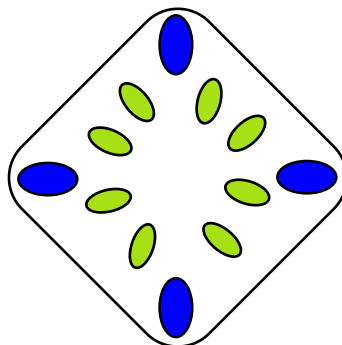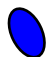

1st order bundle

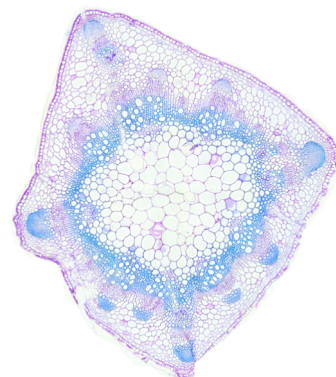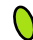

2nd order bundle

Supplement: Supplementary file 5 — Figure S5. Change in bundle number that occurs after rewatering in comparison with a representative control. [file TPJ-106-1338-s005.pdf]

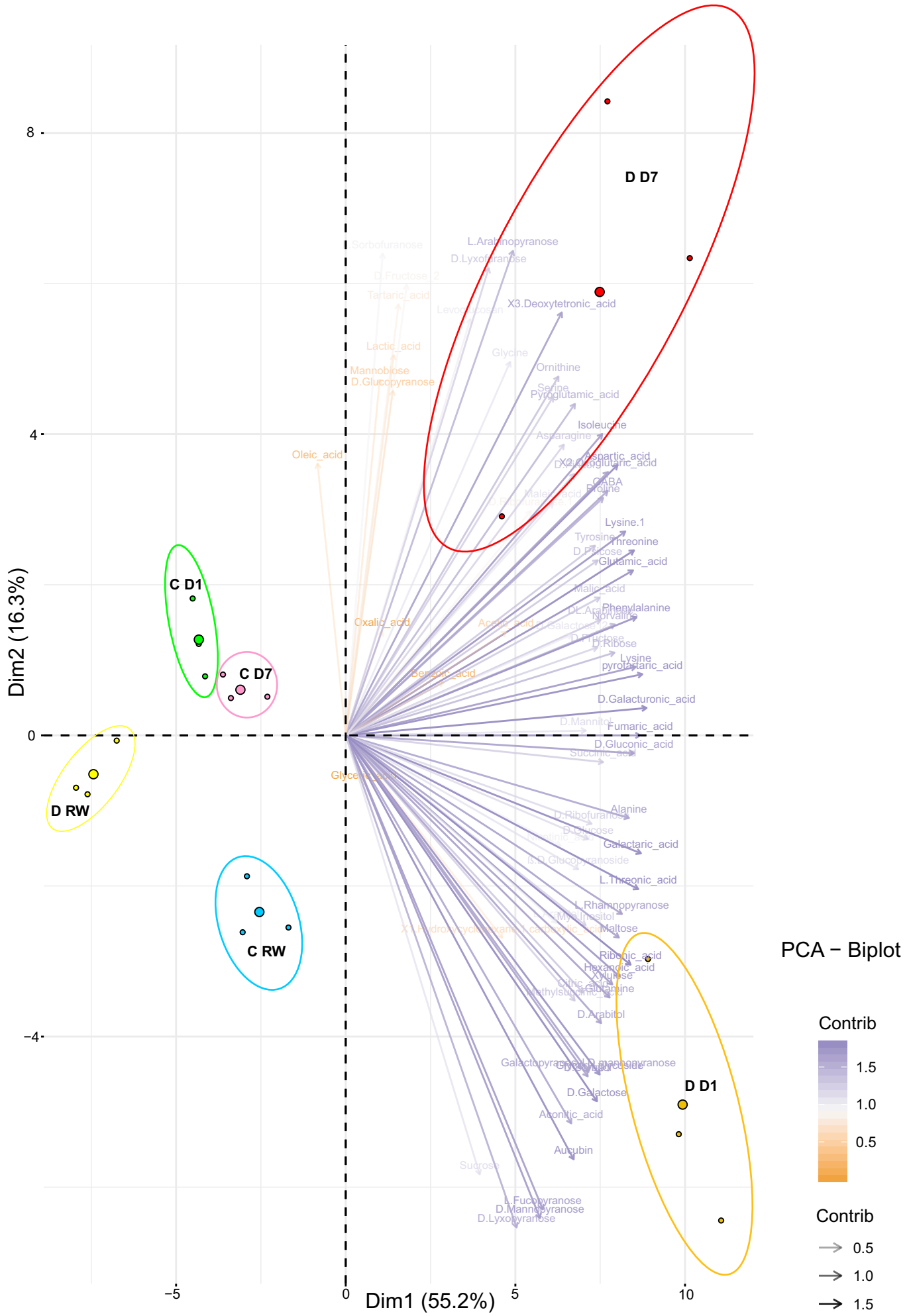

Supplement: Supplementary file 6 — Figure S6. Principal component analysis showing a scatter plot for differentially accumulated metabolites (n = 3) at each time point on principal component 1 (Dim1) and principal component 2 (Dim2). [file TPJ-106-1338-s001.pdf]

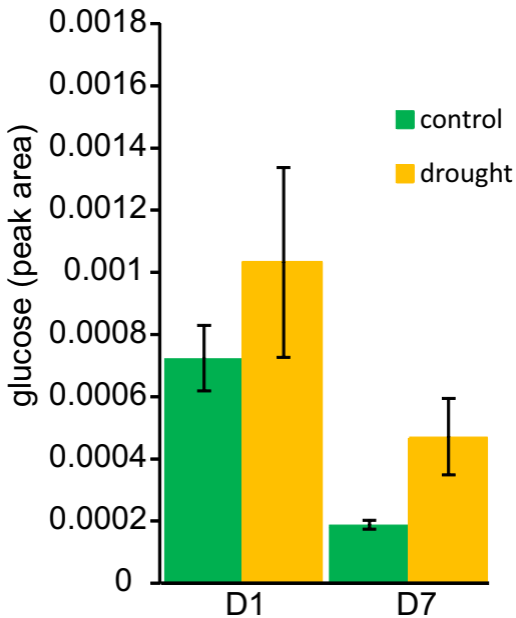

Supplement: Supplementary file 7 — Figure S7. The sum of glucose content in phloem sap collected as separate fractions over short intervals (0, 0.5, 1, 2, 3 and 6 h). [file TPJ-106-1338-s009.pdf]

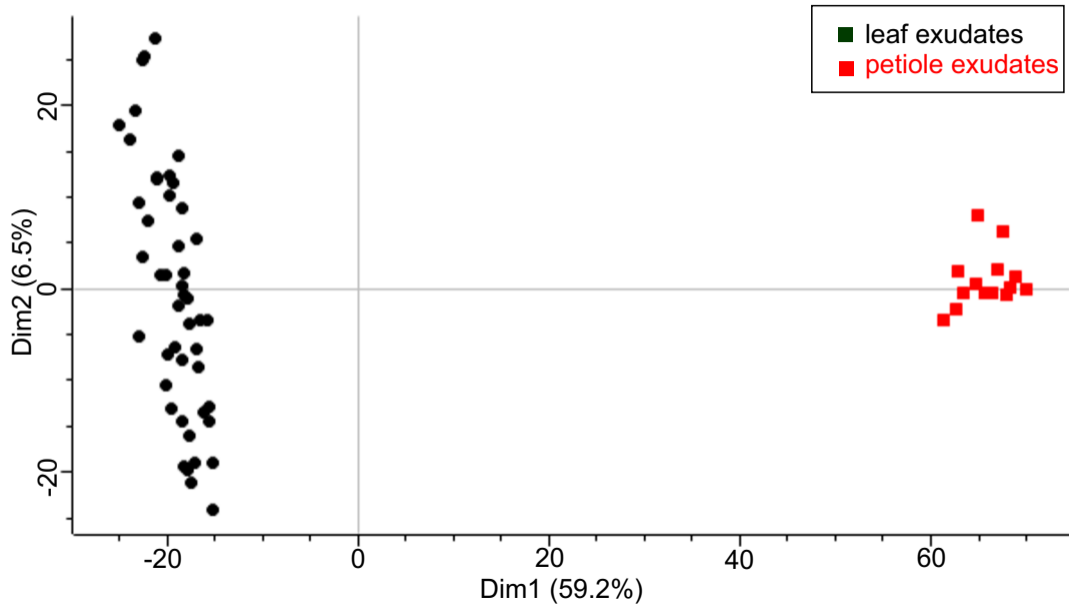

Supplement: Supplementary file 8 — Figure S8. Principal component analysis (n = 3) showing a scatter‐plot comparison of metabolite fingerprints obtained for phloem exudates from leaves (black) and from excised petioles (red). [file TPJ-106-1338-s010.pdf]

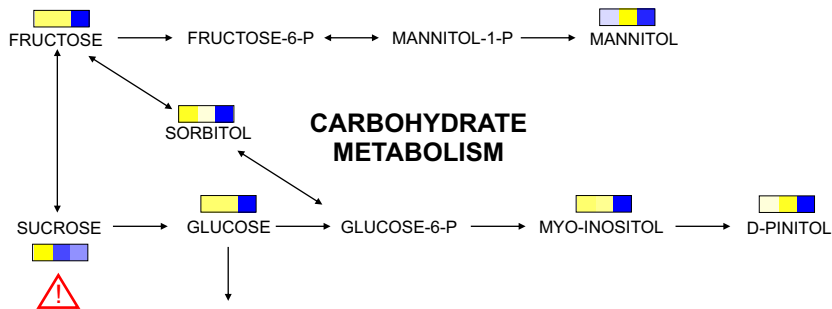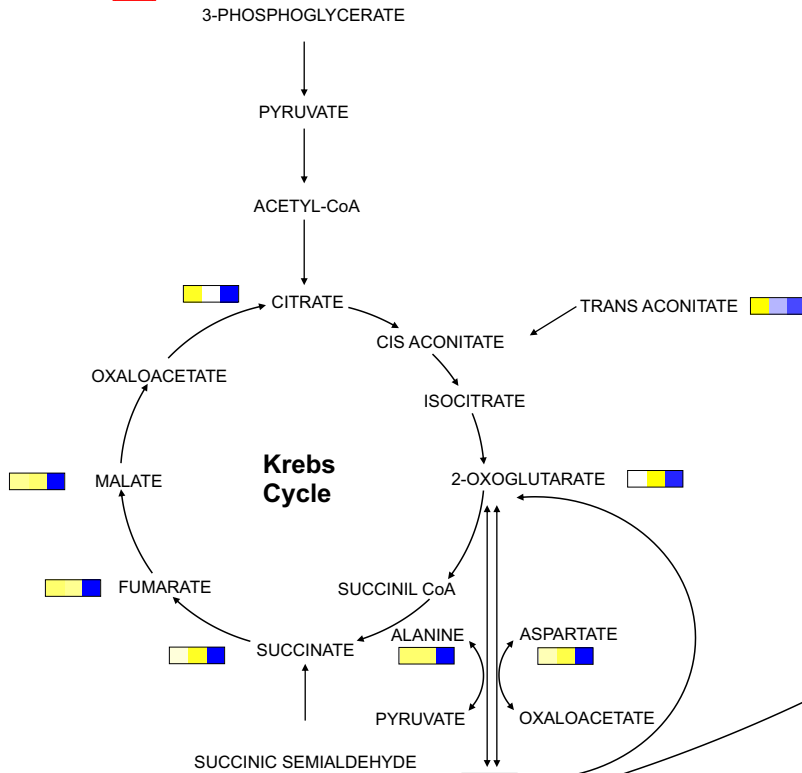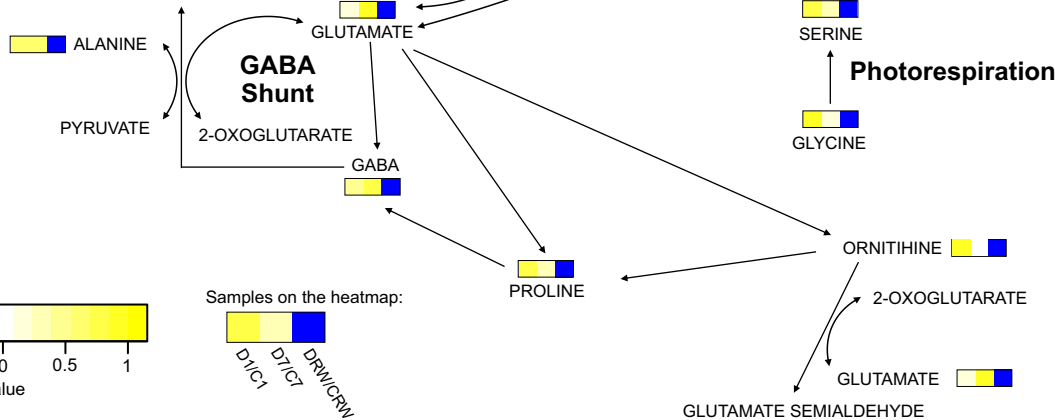

Relative abundance:

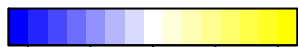

Value

Samples on the heatmap:

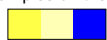

D1/C1  
D7/C1  
DRW/CRW

Supplement: Supplementary file 9 — Figure S9. Visualization of changes in selected metabolites of primary carbon and nitrogen metabolism in phloem exudates of drought‐treated pea plants at the represented times. [file TPJ-106-1338-s004.pdf]
